# A novel extracellular vesicle-associated endodeoxyribonuclease helps *Streptococcus pneumoniae* evade neutrophil extracellular traps and is required for full virulence

**DOI:** 10.1038/s41598-018-25865-z

**Published:** 2018-05-22

**Authors:** Hina Jhelum, Hema Sori, Devinder Sehgal

**Affiliations:** Molecular Immunology Laboratory, National Institute of Immunology, New Delhi, India

## Abstract

*Streptococcus pneumoniae* (pneumococcus) is a major bacterial pathogen that causes pneumonia and septicemia in humans. Pneumococci are cleared from the host primarily by antibody dependent opsonophagocytosis by phagocytes like neutrophils. Neutrophils release neutrophil extracellular traps (NETs) on contacting pneumococci. NETs immobilize pneumococci and restrict its dissemination in the host. One of the strategies utilized by pneumococci to evade the host immune response involves use of DNase(s) to degrade NETs. We screened the secretome of autolysin deficient *S. pneumoniae* to identify novel DNase(s). Zymogram analysis revealed 3 bands indicative of DNase activity. Mass spectrometric analysis led to the identification of TatD as a potential extracellular DNase. Recombinant TatD showed nucleotide sequence-independent endodeoxyribonuclease activity. TatD was associated with extracellular vesicles. Pneumococcal secretome degraded NETs from human neutrophils. Extracellular vesicle fraction from *tatD* deficient strain showed little NET degrading activity. Recombinant TatD efficiently degraded NETs. *tatD* deficient pneumococci showed lower bacterial load in lungs, blood and spleen in a murine sepsis model compared to wildtype strain, and showed less severe lung pathology and compromised virulence. This study provides insights into the role of a novel extracellular DNase in evasion of the innate immune system.

## Introduction

*Streptococcus pneumoniae* (pneumococcus) is a Gram positive extracellular bacterium that causes pneumonia, septicemia, meningitis and otitis media in humans. *S. pneumoniae* is responsible for much morbidity and mortality globally^1,2^. Approximately 14.5 million cases of pneumococcal disease are estimated to have taken place in the year 2,000. The number of HIV negative children <5 years of age who died due to pneumococcal infection were estimated to be 735,000 per year^3,4^.

Several surface exposed virulence determinants such as capsule, pneumolysin, PspA, PsaA among others have been identified in *S. pneumoniae*^1,5^. Bacterial pathogens have devised myriad ways and strategies to evade the host innate and adaptive immune response^6–8^. Pneumococcal virulence factors have been shown to mediate adhesion and/or invasion of the host cells and/or antagonize the immune system. For example, several pneumococcal proteins have been implicated in evasion of complement-mediated immunity^9^.

Pneumococci colonizes the human nasopharynx following inhalation of droplets harbouring *S. pneumoniae*. Pneumococci may stay in the nasopharynx as a commensal or spread to other sites like lung and blood. The first immune cell type to combat *S. pneumoniae* during early infection is the alveolar macrophage^10^. As the infection progresses in the lungs, pneumococcci trigger the recruitment of neutrophils which play a crucial role in the clearance of the bacteria^11^. Neutrophils utilize various strategies like release of peptides with antimicrobial properties and generation of reactive oxygen species to tackle human bacterial pathogens such as *S. pneumoniae*. Pneumococci are phagocytosed by neutrophils and killed by various oxidative and/or non-oxidative mechanisms^12^. Pathogenic microbes are capable of triggering neutrophils to release what are referred to as neutrophil extracellular traps (NETs) by a process called NETosis^13^. NETs comprise of chromatin decorated with various antimicrobial peptides like LL37, neutrophil elastase, myeloperoxidase, defensins among others^14^. NET formation has been reported to be triggered by a variety of pathogens^15,16^. Examples include *Pseudomonas aeruginosa*^17^, *Staphylococcus aureus*^18^ and *Streptococcus pyogenes*^19^. NETs have been demonstrated to be present in lungs of mouse infected with pneumococci^20^.

Bacteria-derived extracellular vesicles (EVs) have been shown to be important in modulating immune response and mediating pathogenesis^21–23^. The size of EVs has been demonstrated to range from 20 to 500 nm^23^. Biochemical and proteomic analysis demonstrated the presence of nucleic acids, lipopolysaccharide, enzymes and virulence factors like toxins that are put to use by the pathogen to initiate and establish disease in the host^22,24–27^. In *S. pneumoniae* EVs have been demonstrated to induce protective immune response^28^. The role of EVs in virulence and their immunomodulatory properties have been studied in bacterial pathogens like *P. aeruginosa* and *Vibrio cholerae*^21^.

Recent studies have elucidated the mechanism employed by bacteria to escape NETs^29^. Wartha *et al*. demonstrated that the polysaccharide capsule protects pneumococci from NETs^30^. Several pathogens have been shown to release nuclease to degrade NETs^31,32^. Several important human pathogens have been documented to produce extracellular DNases which may potentially play a role in pathogenesis, virulence and bacterial fitness. These include *S. aureus*^33^, *S. pyogenes*^34^, *Streptococcus agalactiae*^35^, *Streptococcus sanguinis*^36^, *Haemophilus influenzae*^37^, *Mycobacterium tuberculosis*^38^ and *P. aeruginosa*^39^. *S. pyogenes* is reported to secrete a DNase that aids in pathogenesis by augmenting evasion of innate immune response^40^, other pathogens release extracellular nuclease to degrade the chromatin component of NETs thereby evading the host immune system. Extracellular outer membrane vesicle-associated DNase activity has been reported for the Gram negative bacterium *Porphyromonas gingivalis*^41^. Following degradation of NETs, the pathogen is able to escape and disseminate to the other parts of the host.

Pneumococcal α-enolase has been shown to induce NET formation^42^. A membrane-anchored surface exposed nuclease, EndA (endonuclesae A), was characterised as a DNA entry nuclease^43^. EndA from pneumococcal strain TIGR4 was subsequently shown to degrade NETs^20^. It was observed that EndA-deficient pneumococci exhibited DNase activity albeit at a level lower than observed with the wildtype strain suggesting the presence of additional nuclease(s)^44^. We set out to identify the novel nuclease released by *S. pneumoniae*, and to investigate its potential role in evading the anti-bacterial activity of NETs and establishing infection.

In the present study, we describe the identification, biochemical and functional characterization of a novel nucleotide sequence-independent endodeoxyribonuclease TatD (SPD_1788) from *S. pneumoniae* strain D39. We show that the EV-associated DNase degrades NETs released by activated human neutrophils and its absence compromises the virulence of *S. pneumoniae* in a murine sepsis model. Our data showed that the novel DNase is involved in evading the host innate immune system.

## Materials and Methods

### Bacterial strains and culture condition

The pneumococcal strains ATCC 6301, ATCC 6314 and TIGR4 (ATCC BAA-334) were obtained from American Type Culture Collection, USA. D39 (NCTC 7466) was sourced from National Collection of Type Cultures, United Kingdom. *S. pneumoniae* strain WU2 was a kind gift from Susan K. Hollingshead, University of Alabama, USA. *S. pneumoniae* strains were maintained on tryptic soy agar supplemented with 5% (v/v) sheep blood (TSA) plate or in Todd-Hewitt broth supplemented with 0.5% yeast extract (THY) at 37 °C in the presence of 5% CO_2_ as described previously^45^. Kanamycin (300 µg/ml) and/or erythromycin (10 µg/ml) was supplemented wherever required. For preparing glycerol stocks, mid-logarithmic phase pneumococcal culture was stored in THY with 15% (v/v) glycerol at −70 °C. For some experiments pneumococci were grown in chemically defined medium (CDM)^46^.

*Escherichia coli* strains XL1-Blue and SG13009 were cultured in Luria-Bertani (LB) broth at 220 rpm in an orbital shaker or on LB agar plates supplemented with ampicillin (100 µg/ml) and/or kanamycin (25 µg/ml) at 37 °C.

### Construction of gene deficient mutants in *S. pneumoniae*

Isogenic mutants of *S. pneumoniae* strain D39 deficient in *lytA* (autolysin; *spd_1737*), *endA* (*spd_1762), tatD*, or *endA* and *tatD* (double mutant) were constructed by in-frame replacement of the gene of interest with an antibiotic resistance conferring gene cassette and an overlap PCR-based strategy reported earlier^47,48^. *S. pneumoniae* was transformed with the recombinant construct using competence-stimulating peptide 1 as described by Pozzi *et al*.^49^. The schematic representation of the strategy used for generating and confirming the various pneumococcal mutants is shown in Supplementary Figs S1–S3. The mutants were confirmed by colony PCR, nucleotide sequencing and immunoblotting. The primers used in the construction, confirmation and sequencing of the mutants are listed in Supplementary Table S1.

### Preparation of pneumococcal secretome

*S. pneumoniae* was grown in THY medium (1 litre) till OD_600_ reached 0.6 and cell-free proteins (secretome) were isolated as described by Govindarajan *et al*.^50^. The culture supernatant obtained was passed through a 0.2 μm filter and concentrated to a volume of 10 ml by ultrafiltration using a membrane with a 10 kDa cut off.

### Molecular cloning, expression and purification of recombinant TatD

The gene encoding TatD was PCR amplified from D39 genomic DNA using *Pfu* DNA polymerase (G-Biosciences, India), and DS_1401 and DS_1402 as sense and anti-sense primer, respectively (Supplementary Table S1). The restriction enzyme digested amplicon was cloned in BamHI-PstI digested expression vector pQE-30Xa (Qiagen, Germany) and transformed into *E. coli* strain XL1-Blue. The recombinant DNA construct was validated by restriction analysis and nucleotide sequencing.

For protein expression, the recombinant construct was transformed into *E. coli* strain SG13009 (Qiagen). Recombinant TatD (rTatD) was purified to apparent homogeneity from inclusion bodies as a N-terminal histidine affinity tagged protein using Ni-NTA affinity column chromatography (Qiagen). The purity of recombinant protein was assessed by SDS-PAGE.

### Isolation of pneumococcal extracellular vesicles

Pneumococcal strain D39 or its isogenic mutant deficient in *tatD* was grown in THY medium (1 litre) till the OD_600_ reached 0.5. The EVs were isolated from the secretome as described by Olaya-Abril *et al*.^28^. The protein content of the EV preparation was estimated using a commercially available kit (Thermo Scientific, USA).

### DNase assay

The DNase activity of the pneumococcal secretome and rTatD was assayed as previously described with some modifications^51^. Briefly, the 30 μl reaction mixture consisted of calf thymus DNA (Sigma-Aldrich, USA; 500 ng) and pneumococcal secretome (from *S. pneumoniae* strains D39, D39Δ*lytA*, D39Δ*tatD*, TIGR4, ATCC 6301, ATCC 6314 or WU2; 10 μg) or 10 nM rTatD in DNase buffer (50 mM Tris-HCl/5 mM CaCl_2_, pH 7.2). The sample was incubated at 37 °C for the indicated period of time. Bovine pancreatic DNase I (Sigma-Aldrich) was used as the positive control. EDTA was added to a final concentration of 10 mM and the sample was run on an agarose gel (0.8%) containing ethidium bromide (0.5 μg/ml). The DNA was visualized using a gel documentation system (Gene Genius Bioimaging System, Syngene, UK). In some experiments, calf thymus DNA was substituted with genomic DNA from strain D39 or supercoiled/linear double-stranded plasmid DNA.

In order to test whether the DNase activity was heat labile the secretome was subjected to 65 °C for 20 min. Separately, the secretome was treated with proteinase K (100 μg/ml) at 37 °C for 1 h or with 2.5 mM EDTA for 15 min at 37 °C followed by DNase assay as described above.

### In-gel DNase activity assay

In-gel DNase activity assay (zymogram analysis) was performed using the secretome from D39Δ*lytA* strain or rTatD as described previously with slight modification^52^. Briefly, secretome was resolved on a SDS-PAG containing calf thymus DNA (10 μg/ml). Following electrophoresis, the gel was washed with several changes of water to remove SDS and transferred to renaturation buffer (40 mM Tris/2 mM MgCl_2_, pH 7.7) at 37 °C for 20 h with gentle shaking. The gel was stained with ethidium bromide (0.5 μg/ml) and observed under UV illumination. The DNase activity appeared as dark zones against a fluorescent background of ethidium bromide stained DNA. In parallel, the secretome was resolved on a SDS-PAG (without calf thymus DNA) run under identical conditions and stained with silver nitrate as described by Mortz *et al*.^53^.

### Mass spectrometric and bioinformatic analyses

The silver stained gel slice was processed for mass spectrometric analysis as described previously^54^. The sample was analyzed using 4800 MALDI TOF/TOF analyzer (Applied Biosystems, USA) or EASY-nLC system (Thermo Fisher Scientific, USA) coupled to LTQ Orbitrap-Velos mass spectrometer (Thermo Fisher Scientific). The peptide mixture was resolved using a 10-cm PicoFrit Self-Pack microcapillary column (inner diameter = 75 μm, outer diameter = 360 μm, 15 μm tip) filled with 5 μm C18-resin (New Objective, USA). The analysis was done at the Central Mass Spectrometry facility of the National Institute of Immunlogy (NII).

Bioinformatic analysis was performed on the list of proteins identified by mass spectrometric analysis. The protein sequence was searched for the presence of domains and/or motifs indicative of nucleases. SignalP 4.1 (http://www.cbs.dtu.dk/services/SignalP/) server was used to check for the presence of signal peptide in TatD. TatD sequences from *S. aureus*, *Plasmodium falciparum*, *Trypanosoma brucei, E. coli* and *S. pneumoniae* were aligned using Clustal Omega (https://www.ebi.ac.uk/Tools/msa/clustalo/) and presented using ESPript (http://espript.ibcp.fr/ESPript/ESPript/). Putative structure of TatD was predicted using SWISS-MODEL (https://swissmodel.expasy.org/). MacVector software was used to check for conservation of TatD across pneumococcal strains for which complete genome sequence is available publically (https://www.ncbi.nlm.nih.gov/genome/genomes/176). The allele frequency and percent identity was calculated from this data.

### Immunoblot analysis

Polyclonal antibody was raised against purified rTatD, rLytA and PsaA (SP_1650) in mice using standard procedures (Supplementary Fig. S4). PsaA was purified as a histidine-affinity tagged recombinant protein from *E. coli* expressing the 21 to 309 amino acid sub-fragment of PsaA from pneumococcal strain TIGR4. Pneumococcal whole cell lysates were prepared as described previously^55^. Immunoblot analysis was performed on EV fraction and cell lysates obtained from various pneumococcal strains as described by Sambrook *et al*.^56^. Polyclonal sera against rTatD (diluted 1 in 500), rLytA (diluted 1 in 1,000) or monoclonal antibody against pneumolysin (Cat. No. 63547, Statens Serum Institute, Denmark; diluted 1 in 2,000) was used as the primary antibody. Horseradish peroxidase coupled goat anti-mouse antibody (Cat. No. 554002, BD Bioscience, USA; diluted 1 in 10,000) was used as the secondary antibody. The blots were developed using enhanced chemiluminescence substrate (Thermo Scientific). To confirm equal loading in lanes the blots were reprobed for PsaA.

### Picogreen assay

The DNase activity was quantitated using picogreen assay^57^. The 300 µl reaction mixture consisted of rTatD (10 μM), calf thymus DNA (500 ng) and picogreen dye (Invitrogen, USA). The sample was incubated at room temperature for 5 min followed by addition of 10 mM Tris-HCl/1 mM EDTA, pH 8.0 to make up the volume to 1 ml. The fluorescence of the sample was measured using a spectrofluorometer (Perkin-Elmer, USA) with an excitation and emission wavelength of 480 and 520 nm, respectively. The DNase activity was expressed as the amount of DNA degraded relative to the only DNA control sample expressed in percentage terms. The percent DNase activity was calculated using the formula: DNase activity (%) = 100 – [((fluorescence of only DNA control − fluorescence of treated sample)/fluorescence of only DNA control) × 100]. The effect of addition of divalent cations Ca^2+^, Co^2+^, Mn^2+^, Zn^2+^ and Mg^2+^, and pH on DNase activity of rTatD was also assessed.

### NET degradation assay

Human neutrophils were isolated using the PolymorphPrep system (Axis Shield, Norway) according to manufacturer’s instructions. Briefly, 10 ml blood was withdrawn from healthy human volunteers. Blood and polymorphprep was layered in 1:1 ratio (v/v). The sample was centrifuged at 500 × g for 35 min at 20 °C. Neutrophils were collected from the lower band and washed in Hank’s Balanced Salt Solution without calcium and magnesium (Biological Industries, Israel), resuspended in RPMI and seeded on a poly-L-lysine (Sigma-Aldrich) coated coverslip for 1 h at a density of 4 × 10^5^ cells per ml per well in a 24-well plate as described by de Buhr *et al*.^58^. The neutrophils were stimulated with phorbol 12-myristate 13-acetate (PMA, 25 nM) for 2 h at 37 °C under 5% CO_2_ to release NETs. The NETs were incubated with pneumococci (multiplicity of infection = 1), pneumococcal secretome (10 µg/ml) or rTatD (10 nM) at 37 °C for 1 h. Bovine pancreatic DNase I (1 U/ml) was used as a positive control for NET degradation. The cells were fixed with paraformaldehyde (4%) followed by permeabilization with Triton X-100 (0.5%) for 1 min at room temperature and blocked with bovine serum albumin (2%) for 1 h. NETs were visualized by staining with rabbit anti-myeloperoxidase polyclonal antibody (diluted 1 in 100 in 2% BSA-PBS; Cat. No. ab9535, AbCam, UK) for 1 h at room temperature and DAPI (Invitrogen). Alexa Fluor 488-conjugated goat anti-rabbit IgG antibody (Cat. No. A11008, Invitrogen) was used as the secondary reagent. After washing, Prolong Gold antifade reagent (Invitrogen) was applied to the stained sample.

Images were acquired using a confocal microscope (Leica TCS SP5 II, Leica Microsystems, Germany) with a Plan Apo oil immersion objective (63×; numerical aperture = 1.40). Alexa Fluor 488 and DAPI were excited at 488 and 405 nm, respectively. DFC 360 FX camera (version FCAM2 V1.0.10 Sep1 2009 10:40 FXLib: 5.1.0.10570) was employed to capture images. The images were processed by Leica Application Suite Advanced Fluorescence Lite 2.6.0 build 7266 software. The files were exported from the acquisition software in 8-bit file format. The area of NETs were quantitated using ImageJ software as described by de Buhr *et al*.^58^. Twenty randomly selected frames were analyzed for quantitating degradation of NETs.

### Pneumococcal load in lungs, blood and spleen

To enumerate the pneumococcal load in lungs, blood and spleen following intraperitoneal infection, mice were bled retro-orbitally using EDTA as an anticoagulant. Mice were euthanized by cervical dislocation, and lungs and spleen were harvested. The harvested tissues were homogenized (20,000 rpm; Kinematica AG, Switzerland) in 1 ml chilled sterile PBS. The homogenates were centrifuged at 60 × g for 10 min at 4 °C. The supernatant was collected and centrifuged at 3,300 × g for 15 min at 4 °C. The pellet obtained was resuspended in THY medium containing 15% (v/v) glycerol and stored at −70 °C. The homogenates were serially diluted in sterile PBS and plated on TSA plates.

### Processing, staining and analysis of lung histopathology

Lungs obtained from *S. pneumoniae* infected intraperitoneally or PBS adminstered mice were harvested and fixed in 10% formalin solution for 18 h at 4 °C. The tissue was dehydrated using increasing concentrations of ethanol followed by permeabilization with xylene. The lung piece was embedded in paraffin and sectioned (4 μm) using a microtome (Reichert-Jung, Germany). The sections were stained with haematoxylin-eosin (Sigma-Aldrich) and assessed by a pathologist blinded for the groups. The lung histopathology was analyzed as described previously^59^. Briefly, lung sections were analyzed for intra-alveolar inflammation, interstitial inflammation, bronchitis, edema, endothelialitis and pleuritis. Each parameter was scored on a scale of 0 (absent) to 4 (severe), and total lung inflammation was plotted as histology score. Microscopic images were acquired at a magnification of 100× or 400×.

### Mice survival experiment

Twelve 6–8 week old BALB/c mice per group were infected intraperitoneally with 10^5^ cfu per mouse of D39 or its isogenic strain deficient in *tatD*. The health of the mice was monitored for symptoms every 12 h for 7 days and scored on a scale of moribundity (5 - healthy, normal fur, eyes and activity; 4 - beginning to look sick and ruffled coat; 3 - sick, ruffled coat and decreased activity; 2 - very sick, ruffled coat and secretion from eyes; 1 - extremely sick, near death, little or no activity and decreased breathing). Animals were euthanized at the end of the experiment or when they turned moribund (score = 1).

### Ethics statement

All recombinant DNA work was done with the approval, and according to the guidelines and regulations laid out by the NII Institutional Biosafety Committee (IBSC#62/02/09/12). All mice experiments were performed with the approval and in accordance with the guidelines and regulations of the NII Institutional Animal Ethics Committee (IAEC#327/113). All experiments that required the use of human blood were performed with the approval and in accordance with the relevant guidelines and regulations of the NII Institutional Human Ethics Committee (IHEC#78/13). Informed consent was obtained from all participants. The inclusion criteria used were: the human volunteers should be healthy, be between the age of 20–50 years and should weigh more than 50 kg. Individual who had a history of infection or individuals who received antibiotics during the preceding 4 weeks were excluded from consideration.

### Statistical analysis

All experiments were done 2-3 times with 2-3 technical replicates. Statistical analysis was performed using GraphPad Prism software version 6 (GraphPad Software Inc., USA). The biochemical data regarding the effect of divalent cations on DNA degrading activity of rTatD was analyzed by one-way ANOVA (with Dunnett’s multiple comparison test). The DNase activity in the EV fractions, bacterial load, and quantitation of lung histopathology and relative expression of transcripts were analyzed by unpaired two-tailed Student’s *t* test. For experiments involving NET degradation, significant outliers were excluded using ROUT’s test, and D’Agostino and Pearson omnibus normality test was used to confirm normal distribution of the data prior to analyzing it using unpaired two-tailed Student’s *t* test or one-way ANOVA (with Dunnett’s multiple comparison test). Kaplan-Meier survival data from the intraperitoneal and intranasal infection models was analyzed by log-rank (Mantel-Cox) test. *p* values of <0.05 were considered statistically significant.

## Results

### Pneumococcal secretome exhibits DNase activity

**Figure 1:**
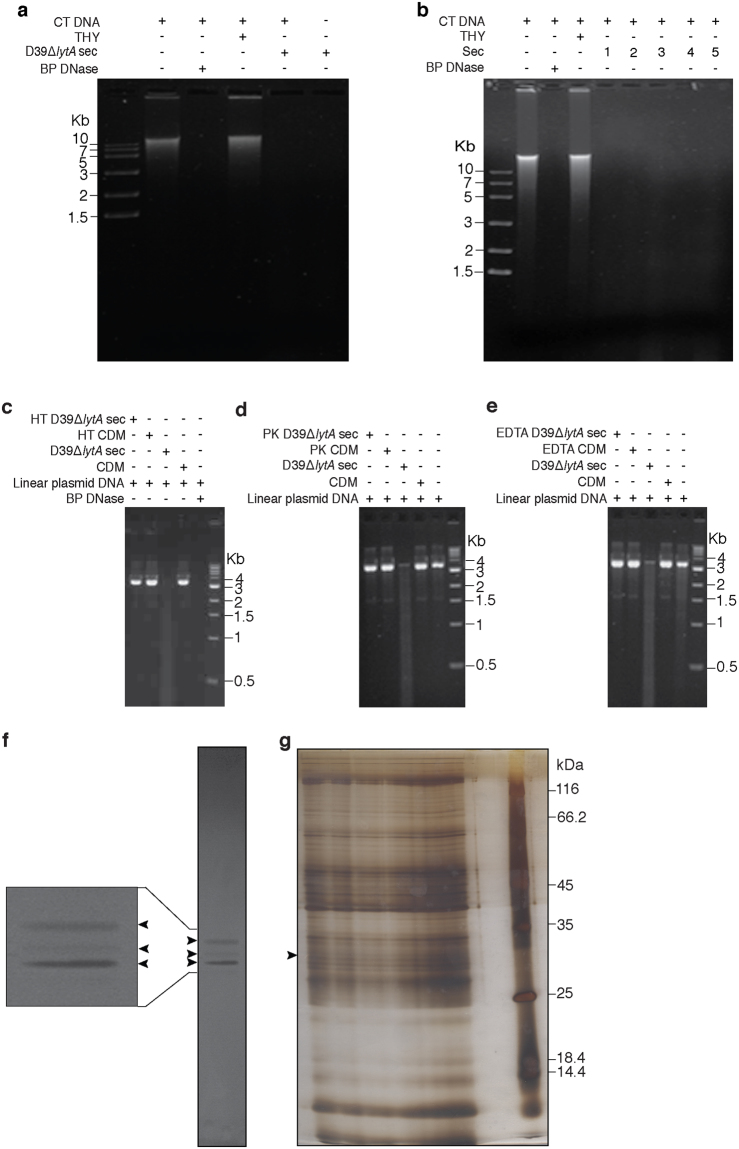
Pneumococcal secretome possesses DNase activity. (**a**) Calf thymus DNA (CT DNA) was incubated in the presence or absence of secretome from *lytA* deficient mutant of *S. pneumoniae* strain D39 grown in THY medium (D39Δ*lytA* sec). The samples were incubated at 37 °C for 30 min. The processed samples were resolved on an ethidium bromide stained agarose gel (0.8%; uncropped image). Bovine pancreatic DNase I (BP DNase) was used as a positive control. Secretome only and THY medium was taken as negative and vehicle control, respectively. (**b**) DNase activity of the secretome (sec) derived from pneumococcal strains D39 (1; served as the positive control), TIGR4 (2), ATCC 6301 (3), ATCC 6314 (4) and WU2 (5). The DNase activity observed with the secretome obtained from D39Δ*lytA* propagated in chemically defined medium (CDM) is heat labile (**c**), and sensitive to treatment with proteinase K (**d**) and EDTA (**e**). Linear double-stranded plasmid DNA was used as the substrate in panels c-e. The molecular mass marker (in Kb) is shown. Heat, proteinase K and EDTA treatment is indicated by ‘HT’, ‘PK’ and ‘EDTA’, respectively. (**f**) Secretome from D39Δ*lytA* was resolved on SDS-PAG bearing calf thymus DNA (30 μg/ml) followed by ethidium bromide staining as described in the *Materials and Methods*. Three dark bands corresponding to DNA degradation were observed against fluorescent background (indicated using arrowheads). A portion of the gel has been enlarged to highlight the three bands. (**g**) The bands corresponding to the three zones of DNA degradation were excised from a silver stained preparative polyacryamide gel, tryptic digested and subjected to mass spectrometric analysis. The molecular mass marker (in kDa) is shown on the right. The full-length gels for panels b-g can be viewed in Supplementary Fig. S7.

Many bacterial pathogens have been shown to secrete/release nuclease(s). In order to check for the presence of DNase(s) in the secretome, we constructed and characterised a *lytA* deficient mutant of the pneumococcal strain D39 by in-frame gene replacement mutagenesis (Supplementary Fig. S1). The rationale for using *lytA* deficient mutant is as follows. LytA is involved in pneumococcal autolysis. Pneumococcal cells are resistant to lysis during exponential phase of the growth curve but are sensitive during stationary phase^60^. In order to minimize potential cytoplasmic proteins from contaminating our secretome preparations (i) we deleted *lytA* gene from the pneumococcal genome and (ii) took *lytA* deficient pneumococcal cultures in mid-logarithmic phase for isolating the secretome. Secretome from D39Δ*lytA* strain degraded calf thymus DNA indicating the presence of DNase activity (Fig. 1a).

To test whether the DNase activity is conserved across pneumococcal serotypes, secretome from strains D39 (serotype 2; positive control), TIGR4 (serotype 4), ATCC 6301 (serotype 1), ATCC 6314 (serotype 14) and WU2 (serotype 3) was subjected to DNase assay (Fig. 1b). We observed that secretome of all the strains tested degraded DNA suggesting that the DNase activity is conserved across various pneumococcal serotypes. Functional characterization of secretome revealed that the DNase activity was heat labile, and sensitive to treatment with proteinase K and EDTA (Fig. 1c–e).

### TatD is a novel putative DNase present in the secretome

In order to identify the DNase(s) in the pneumococcal secretome, in-gel DNase activity assay (zymogram analysis) was done. The data revealed the presence of 3 zones of clearance suggestive of DNase activity (Fig. 1f). The middle band appears to be lighter than the other two bands. The three bands corresponding to DNA degradation were excised, tryptic digested and subjected to mass spectrometry. The mass spectrometric analysis of the middle band (Fig. 1g) revealed the presence of 39 proteins (Supplementary Table S2). The analysis of the topmost and the bottommost bands (Fig. 1f) are subject of independent studies. The identified proteins were further analyzed based on the correct molecular mass range (25 to 35 kDa), literature survey and presence of domains and/or motifs indicative of DNase activity. Based on these analyses TatD was identified as a potential candidate DNase.

### Bioinformatic analysis of TatD

**Figure 2:**
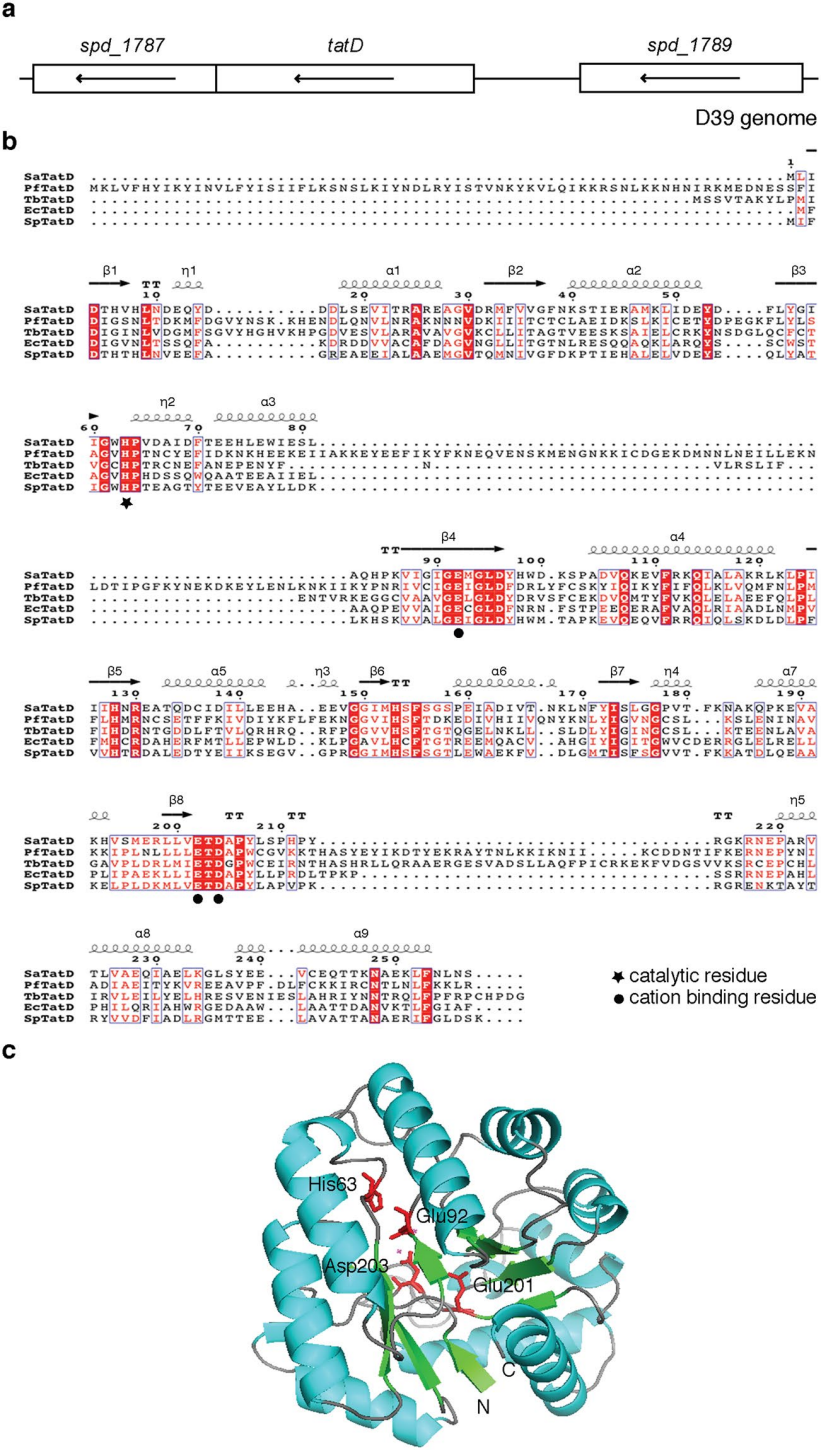
Protein sequence analysis and prediction of the three dimensional structure of TatD. (**a**) *tatD* is flanked by *spd_1787* (ribonuclease M5) and *spd_1789* (cell wall surface anchor protein). Arrow in the rectangular box indicates the transcriptional orientation of gene in the annotated D39 genome. The diagram is drawn to scale. (**b**) Protein sequence alignment of TatD from *S. pneumoniae* (SpTatD) using ESPript with its homologues from *S. aureus* (SaTatD), *P. falciparum* (PfTatD), *T. brucei* (TbTatD) and *E. coli* (EcTatD). Amino acids highlighted in blue open boxes indicate similar residues whereas those highlighted in red filled boxes indicate identical residues. Asterisk and filled circle indicates conserved catalytic and cation binding residues, respectively. Long squiggles indicate alpha helices, arrows indicate beta strands, η indicates 3–10 helix and TT letters represents strict β-turns. Gap in the sequence is indicated by a dot (.). The predicted alpha helices and beta sheets are numbered. (**c**) Structural modeling of TatD using SWISS-MODEL. The best match was found to be with *S. aureus* TatD (PDB ID: 2GZX). Alpha helices, beta-sheets and loops are shown by cyan, green and brown colour, respectively. Catalytic (His63) and cation binding (Glu92, Glu201 and Asp203) residues in TatD are highlighted in red.

The gene immediately to the left of *tatD* in the genome of pneumococcal strain D39 encodes 5S rRNA maturation endonuclease (ribonuclease M5, SPD_1787; Fig. 2a). The gene immediately to the right of *tatD* codes for a cell wall surface anchor protein (SPD_1789). A search of the Pfam database using TatD as the query sequence indicated that it belongs to the amidohydrolase superfamily (CL0034); also referred to as superfamily of metallo-dependent hydrolases. The family shows conservation in tertiary structure (TIM barrel fold) and in the active site residues. More specifically it is a member of the TatD_DNase (PF01026) family. EcTatD (from *E. coli*), a member of this family, has been shown experimentally to have DNase activity^61^. Analysis done using the SignalP 4.1 prediction tool suggested that TatD lacks a signal peptide.

A multiple sequence alignment of TatD with the amino acid sequence of its homologues from *S. aureus*, *P. falciparum*, *T. brucei* and *E. coli* using Clustal Omega is shown in Fig. 2b. The amino acid sequence of TatD from *S. pneumoniae* was used as query sequence in SWISS-MODEL to identify potential structural homologues. The top hit was TatD protein from *S. aureus* (Global Model Quality Estimation score = 0.80 and Qualitative Model Energy Analysis score = −0.01). Based on the sequence analysis it can be predicted that His63 is the catalytic residue, and Glu92, Glu201 and Asp203 are involved in binding divalent cations (Fig. 2b,c).

We next looked at sequence conservation of TatD within and in related species. It was observed that TatD was present in all the 33 pneumococcal strains for which whole genome sequence is available at NCBI suggesting that it plays an important role in pneumococcal biology. Strain R6 was omitted from the analysis as it is a derivative of D39 (which was included in the analysis). Our analysis suggests that TatD is a highly conserved protein. Sequence analysis indicate that TatD exists as (at least) 16 alleles (Supplementary Table S3) with an inter-allelic amino acid sequence identity ranging from 98.05–100%. The relative allele frequency varied from 3.03 to 18.18% corresponding to 1 to 6 alleles. Homologues of TatD are also highly conserved in related species such as *Streptococcus oralis* subspecies *dentisani*, *Streptococcus mitis* and *Streptococcus infantis* with an amino acid identity of 96.50% (248 out of 257), 96.50% (248 out of 257) and 92.22% (237 out of 257), respectively.

### Cloning, expression and purification of rTatD

**Figure 3:**
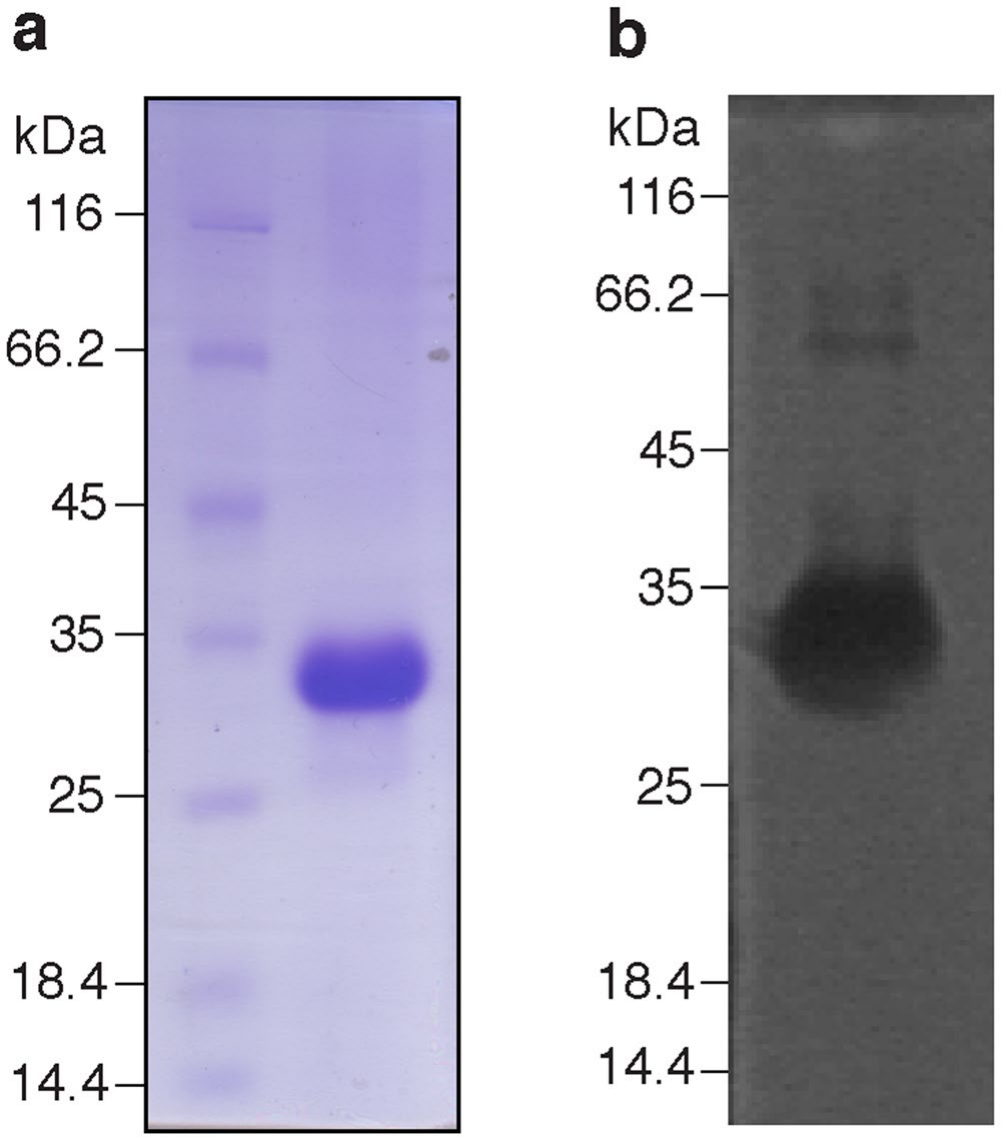
Purification and in-gel DNase activity of rTatD. (**a**) SDS-PAGE analysis of purified recombinant TatD (rTatD). (**b**) In-gel DNase activity with rTatD (5 μg/ml). A dark zone of DNA degradation was observed against fluorescent background. The molecular mass marker (in kDa) is shown on the left of each panel. The full-length gels are shown in Supplementary Fig. S7.

The open reading frame encoding *tatD* was molecularly cloned in the *E. coli* expression vector pQE30-Xa. rTatD (31.8 kDa) was expressed as a N-terminal histidine affinity tagged protein and purified to apparent homogeneity by nickel affinity chromatography from inclusion bodies as assessed by SDS-PAGE (Fig. 3a). The yield of rTatD was estimated to be 40 mg per litre of culture. The identity and molecular mass of rTatD was confirmed by mass spectrometry. In-gel DNase activity assay showed that rTatD has DNA degrading activity (Fig. 3b).

### Biochemical analysis of rTatD

**Figure 4:**
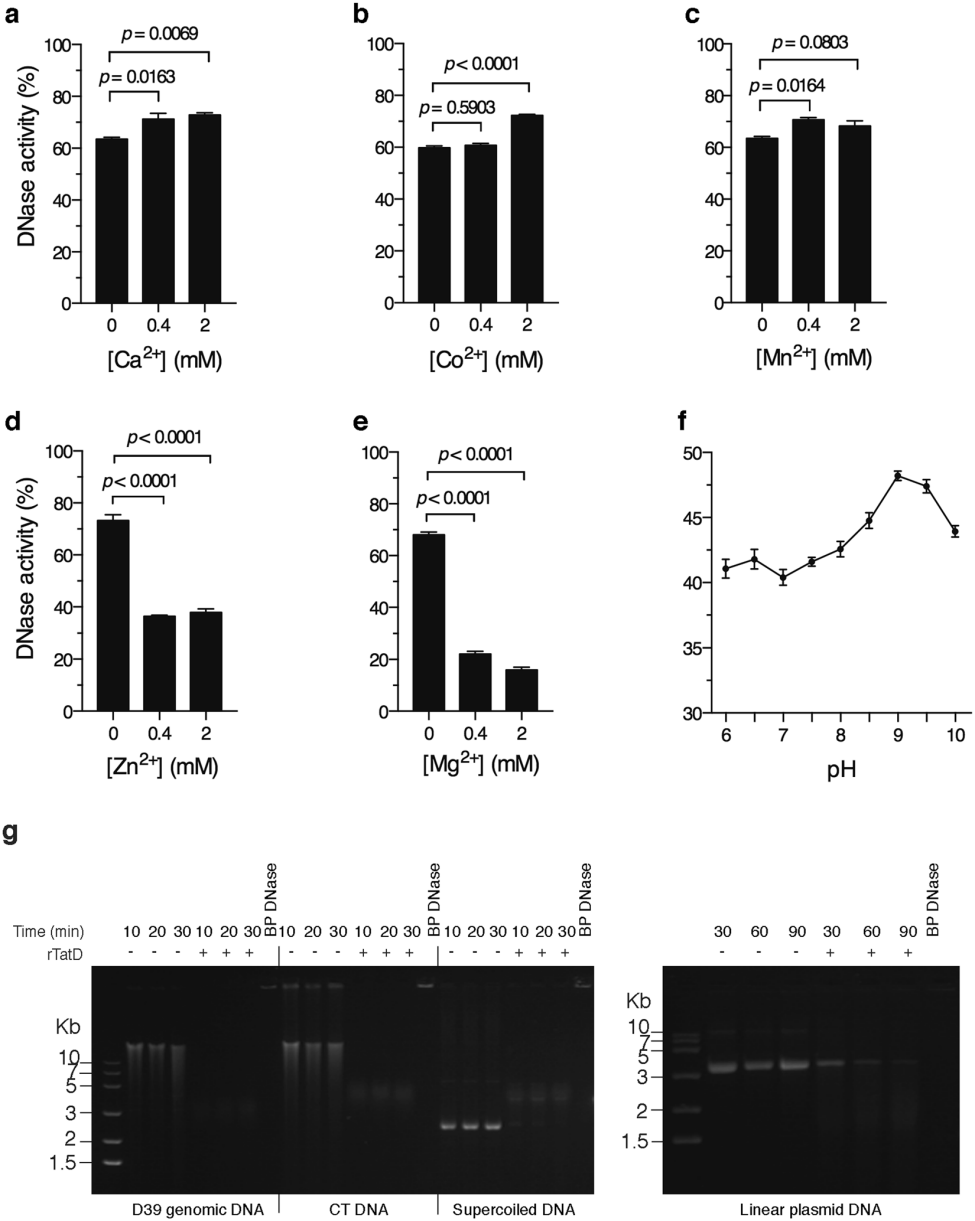
Biochemical characterization of rTatD. (**a**–**e**) Analysis of the effect of presence or absence of various divalent cations on DNase activity of rTatD by picogreen assay. The reaction was performed in 40 mM Tris-HCl (pH 7.7) in the absence or presence of 0.4 or 2 mM Ca^2+^ (**a**), Co^2+^ (**b**), Mn^2+^ (**c**), Zn^2+^ (**d**) or Mg^2+^ (**e**). The DNase activity is represented as mean ± sem of triplicates. The experiment was done twice and data from a representative experiment is shown. One-way ANOVA (with Dunnett’s multiple comparison test) was used for statistical analysis of data presented in panels a-e. (**f**) Effect of pH on DNase activity of rTatD. The reaction was performed in the same reaction buffer as used in DNase assay but with the pH ranging from 6 to 10. The DNase activity was estimated using picogreen assay. Error bars represent mean ± sem of triplicates. A representative of three independent experiments is presented. (**g**) The ability of rTatD to digest DNA substrates of different topologies was checked at the indicated time points. Genomic DNA from pneumococcal strain D39, calf thymus DNA (CT DNA), supercoiled or linearized double-stranded plasmid DNA was incubated with 10 μM rTatD for the indicated time duration. The samples were resolved on an agarose gel containing ethidium bromide. Bovine pancreatic DNase I (BP DNase) was used as the positive control. A representative of three independent experiments is shown. The full-length gels are presented in Supplementary Fig. S7.

The effect of absence or presence of divalent cations on the DNase activity of rTatD was analyzed using picogreen assay. rTatD was incubated with calf thymus DNA for 30 min at 37 °C before adding EDTA. The undigested DNA was stained with picogreen dye. The observed fluorescence was used to calculate the DNase activity. rTatD showed some (“basal”) activity in the absence of any exogenously added divalent cation. The DNase activity was enhanced to a slight but statistically significant extent in the presence of Ca^2+^, Co^2+^ and Mn^2+^ (Fig. 4a–c). Addition of Zn^2+^ and Mg^2+^ inhibited the DNase activity (Fig. 4d,e).

To determine the dependence of DNase activity on pH, rTatD was incubated with calf thymus DNA in a buffer with pH ranging from 6 to 10. rTatD showed DNase activity across a wide pH range with the maximum activity at pH 9 (Fig. 4f).

We next tested for DNase activity as a function of the nature of DNA substrate. rTatD degraded pneumococcal genomic DNA, calf thymus DNA and supercoiled double-stranded plasmid DNA in as little as 10 min but it took more than an hour to digest linearized double-stranded plasmid DNA (Fig. 4g). The fact that rTatD can digest supercoiled double-stranded plasmid DNA suggested that it has an endonuclease activity. These data indicate that TatD is a non-specific endodeoxyribonuclease.

### TatD is associated with the extracellular vesicle fraction

**Figure 5:**
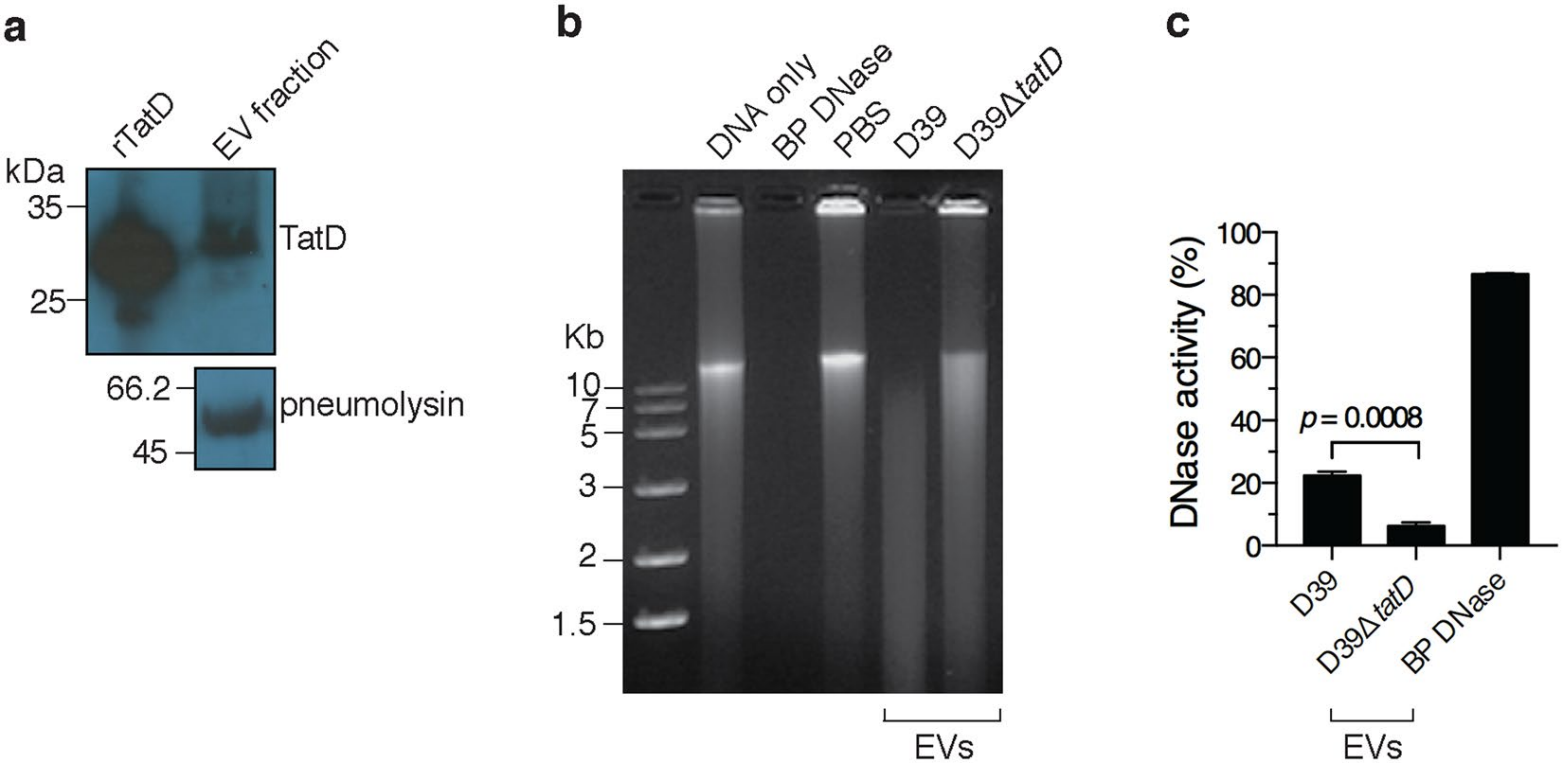
TatD is associated with pneumococcal extracellular vesicle fraction. (**a**) The extracellular vesicle (EV) fraction from *S. pneumoniae* strain D39 was immunoblotted with mouse anti-rTatD polyclonal antibody. We probed for pneumolysin as a positive control as pneumolysin (53 kDa) has previously been demonstrated to be present in the EV fraction. This experiment was done three times. Data from a representative experiment is shown. (**b**) DNase assay was performed with EVs isolated from D39 and D39Δ*tatD* strains as described in the *Material and Methods*. Bovine pancreatic DNase I (BP DNase) and PBS served as the positive and negative control, respectively. This experiment was performed thrice and data from a representative experiment is presented. The full-length blot (panel a) and gel (panel b) can be viewed in Supplementary Fig. S7. (**c**) The DNase activity present in the EV fraction (1 μg) from D39 and D39Δ*tatD* strains was quantitated using picogreen assay. The experiment was performed twice. The data from a representative experiment is shown. Two tailed unpaired *t* test was performed and error bars represent mean ± sem of duplicates.

As indicated previously bioinformatic analysis suggested that TatD lacks a signal peptide. The presence of signal peptide-less proteins in the EV fraction has been reported recently^28^. In order to explore this possibility we isolated EVs from strain D39 using Optiprep density gradient centrifugation. We confirmed the presence of TatD in the EV fraction by immunoblotting with polyclonal serum raised against the recombinant protein (Fig. 5a). We used pneumolysin as a control as it has previously been shown to be present in the EV fraction^28^. DNase assay was performed with EVs isolated from D39 and D39Δ*tatD* pneumococci. EVs from D39 strain degraded calf thymus DNA whereas EVs from D39Δ*tatD* showed little DNA degrading activity (Fig. 5b). The DNase activity present in the EV preparation from wildtype and *tatD* deficient strain was quantitated using picogreen assay (Fig. 5c). The DNase activity in the EV fraction from D39Δ*tatD* (6.27 ± 1.11%) is 28.12% of that observed with EV fraction from wildtype pneumococci (22.30 ± 1.33%). These observations suggest the association of TatD with the EV fraction.

### Secretome and extracellular vesicles from *S. pneumoniae*, and rTatD degrades NETs

**Figure 6:**
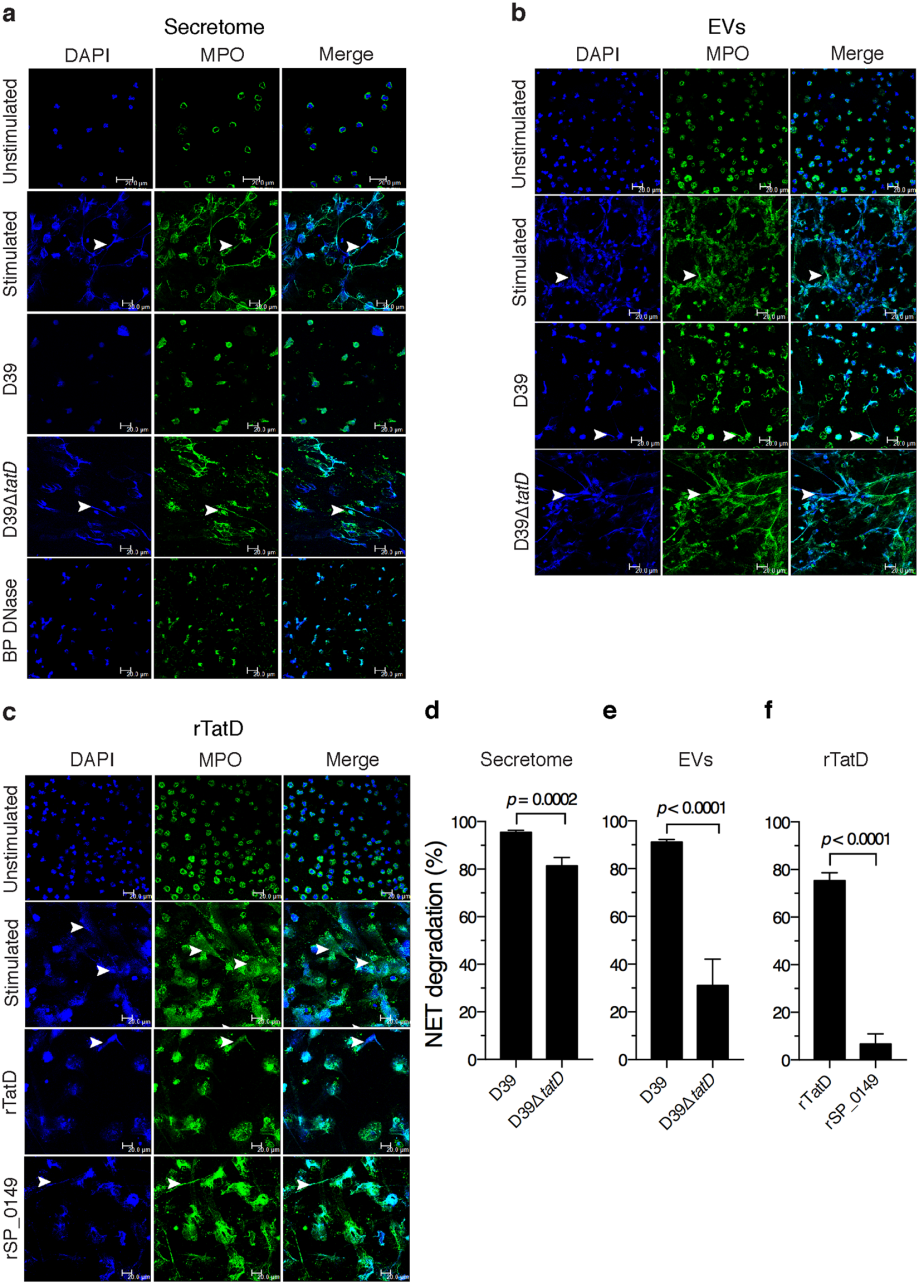
Degradation of NETs by pneumococcal secretome, EVs and rTatD. Human neutrophils were treated with 25 nM PMA for 2 h to release NETs (stimulated). NETs were incubated with (**a**) secretome from D39 or D39Δ*tatD* (10 μg/ml). Bovine pancreatic DNase I (BP DNase) was used as the positive control. NETs were visualized using a confocal microscope following staining with DAPI (blue) and anti-myeloperoxidase antibody (MPO, green). (**b**) NETs incubated with EVs from D39 or D39Δ*tatD*. (**c**) NETs treated with rTatD. A non-relevant pneumococcal recombinant protein (rSP_0149) was used as a negative control. Scale bars represent 20 micron. Arrowheads are used to indicate NETs. (**d**–**f**) Twenty microscopic fields were selected at random for quantitative analysis using ImageJ software. NET degradation is represented as the fraction of NETs present in the treated sample as compared to PMA stimulated sample (control) expressed in percentage terms. Two tailed unpaired *t* test was used for statistical analysis. Error bars represent mean ± sem. Each experiment was performed twice and data from a representative experiment is shown. All the images shown in panels a-c are uncropped.

NETs released from PMA stimulated human neutrophils were incubated with pneumococcal secretome, EVs from strains D39 and D39Δ*tatD* or rTatD. NETs were visualized by staining with DAPI and a myeloperoxidase specific antibody, and degradation was quantitated (Fig. 6). The secretome from D39Δ*tatD* degraded NETs (81.4 ± 3.52%) to a lesser extent compared to secretome from D39 (95.50 ± 0.80%; Fig. 6a,d). The degradation of NETs for EVs prepared from D39 and D39Δ*tatD* strain was 91.1 ± 1.12 and 31.1 ± 11.0%, respectively (Fig. 6b,e). rTatD efficiently degraded NETs (75.4 ± 3.27%; Fig. 6c,f). An irrelevant recombinant pneumococcal protein (rSP_0149) showed little NET degradation activity (6.69 ± 4.24%). Taken together, these data suggest TatD plays a role in degradation of NETs.

### TatD deficiency compromises the capacity of pneumococci to degrade NETs

**Figure 7:**
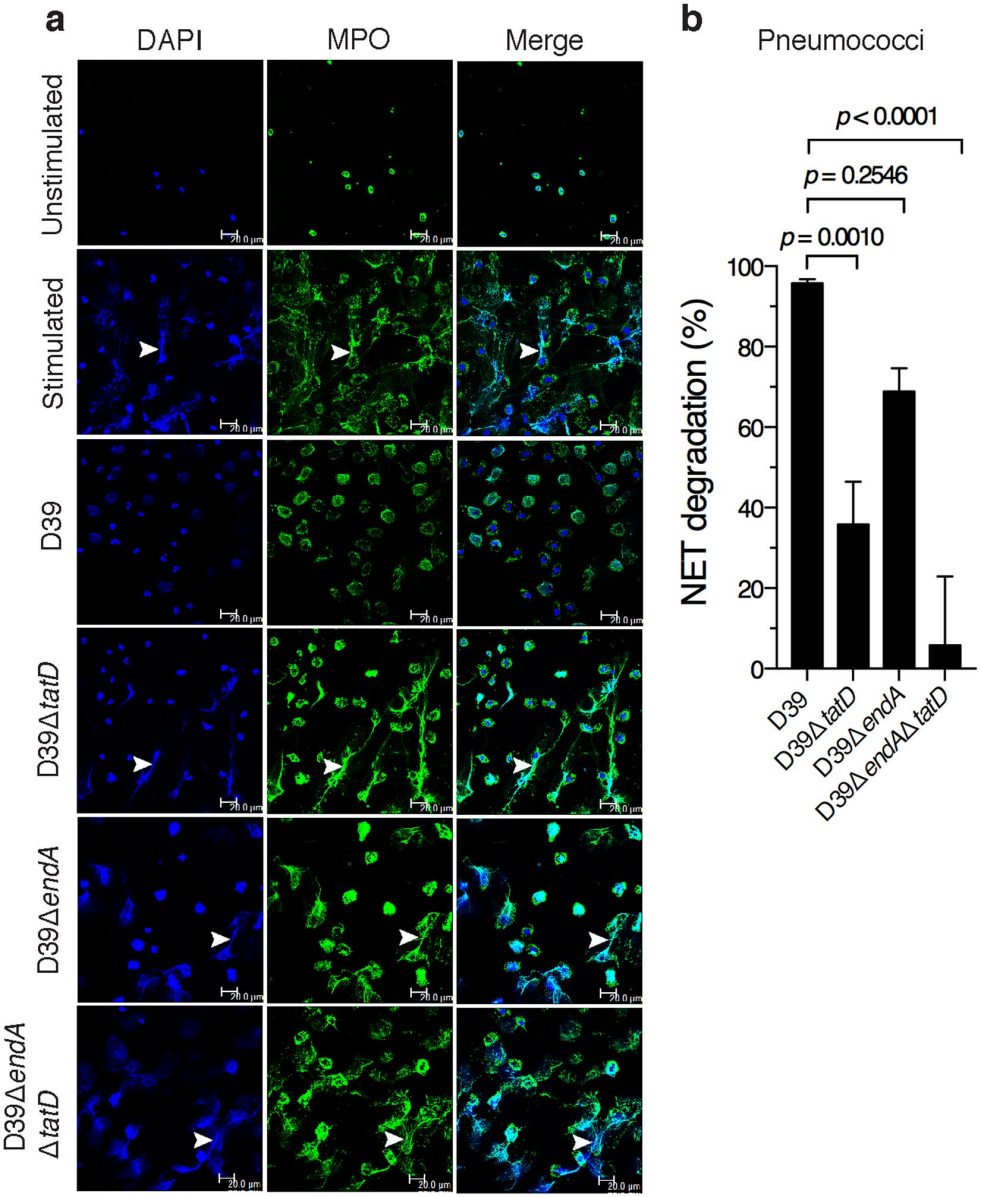
TatD deficiency impairs the ability of pneumococci to degrade NETs *in vitro*. (**a**) Human neutrophils were stimulated with PMA (25 nM) for 2 h to release NETs. This was followed by treatment with cytochalasin D (to prevent phagocytosis). The cytochalasin D treated samples were incubated with D39, D39Δ*tatD*, D39Δ*endA*, D39Δ*endA*Δ*tatD* pneumococci at a multiplicity of infection of 1 for 1 h. NETs were visualized as described in the legend to Fig. 6. All the images shown in panel a are uncropped. (**b**) The images were scored and quantitated as described in *Materials and Methods*. Error bars are represented as mean ± sem. A representative of two independent experiments is presented. The data was analysed statistically using one-way ANOVA (with Dunnett’s multiple comparison test). None of the images in panel a have been cropped.

We investigated the role of TatD in the degradation of NETs using live pneumococci. NETs were incubated with D39, D39Δ*tatD*, D39Δ*endA* or D39Δ*endA*Δ*tatD* pneumococci. We included EndA in this analysis as the membrane anchored endonuclease has been shown to contribute to NET degradation^20^. The ability to degrade NETs was impaired in D39Δ*tatD* mutant (35.8 ± 10.6%) compared to the wildtype D39 strain (95.7 ± 1.01%). The extent of NET degradation observed with D39Δ*endA* strain was lower but not statistically significant compared to the wildtype D39 strain (68.8 ± 5.77 versus 95.7 ± 1.01%; Fig. 7). The deletion of one nuclease can theoretically result in increased expression of the second nuclease to compensate for the loss of the former. To rule out that there is no large difference in the levels of *tatD* and *endA* transcripts in the single mutants D39Δ*endA* and D39Δ*tatD* in comparison to the wildtype D39 strain semi-quantitative reverse-transcriptase PCR was carried out (Supplementary Fig. S5). Our data suggests that *tatD* transcript level was comparable in D39 and D39Δ*endA*. Similarly, the transcript level of *endA* was comparable in wildtype and *tatD* deficient strain. In other words, the loss of *tatD* gene did not result in elevated expression of *endA* transcripts in D39Δ*tatD* mutant. Similarly, the deletion of *endA* gene did not lead to increased expression of *tatD* transcript in D39Δ*endA* strain. In this analysis the constitutively expressed housekeeping gene *gyrB* (DNA gyrase B subunit; *spd_0709*) was used as an internal reference. The deletion of both *tatD* and *endA* seems to have some additive effect which severely compromised the ability of pneumococci to degrade NETs. The relative contribution of TatD towards degradation of NETs appears to be higher compared to that of EndA. Collectively, these data suggest a possibility that TatD plays a crucial role in NET degradation *in vitro*.

### *S. pneumoniae* deficient in *tatD* is compromised in virulence

**Figure 8:**
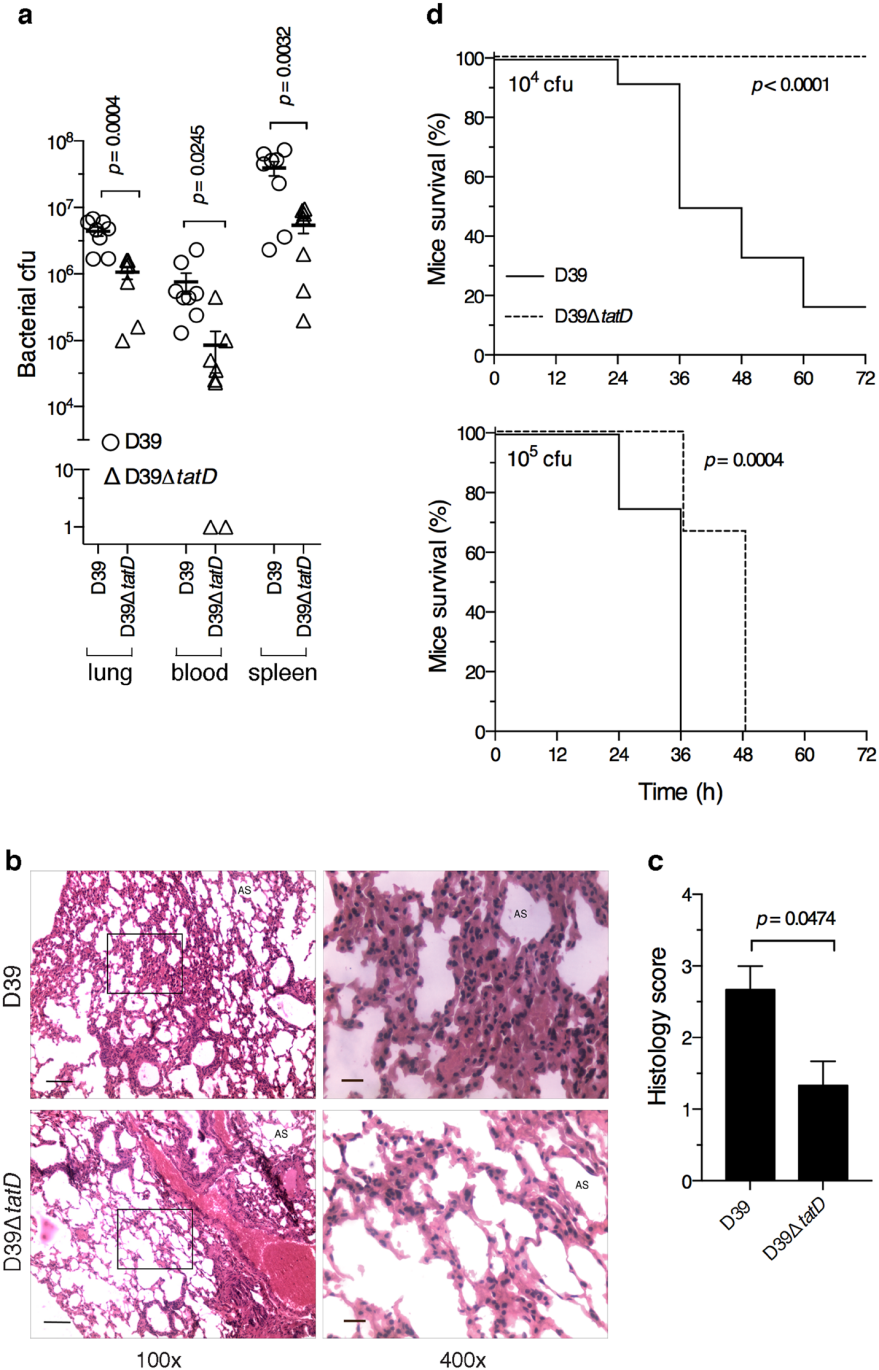
Loss of *tatD* compromises pneumococcal virulence in mice. (**a**) Pneumococcal load in lung, blood and spleen was assessed at 24 h time point in mice infected intraperitoneally with D39 or D39Δ*tatD* (8 mice per group). The lung was processed for enumerating bacteria by dilution plating method. Two tailed unpaired *t* test was used for statistical analysis. The horizontal bar represents mean of triplicates. The experiment was done twice. (**b**) A representative haematoxylin-eosin stained lung section from mice infected intraperitoneally with D39 or D39Δ*tatD* pneumococci 24 h post infection. The magnification of the pictures on the left is 100×. An enlarged portion (boxed) is shown on the right (magnification = 400×). The scale bar represents 100 and 20 micron for pictures with 100× and 400× magnification, respectively. ‘AS’ indicates alveolar spaces. None of the four images in panel b have been cropped. (**c**) Histology scores indicating total lung inflammation are plotted. Data is presented as mean ± sem (3 mice per group). (**d**) Kaplan-Meier survival analysis of BALB/c mice (12 per group) infected intraperitoneally with 10^4^ (upper panel) or 10^5^ (lower panel) cfu per mouse of *S. pneumoniae* D39 or D39Δ*tatD* strain. The health of mice was monitored and scored as described in the *Material and Methods*. Statistical analysis was done using Log-rank (Mantel-Cox) test.

In order to understand the role of TatD in pneumococcal virulence BALB/c mice were infected intraperitoneally with either D39 or D39Δ*tatD* pneumococci, and the bacterial load was enumerated 24 h post infection. The bacterial load in mice infected with wildtype pneumococci was higher in lung, blood and spleen compared to mice infected with *tatD* deficient isogenic strain (Fig. 8a). The pneumococcal load in the lung, blood and spleen of mice infected with D39 was 4.39 ± 0.69 × 10^6^, 7.64 ± 2.64 × 10^5^ and 3.92 ± 0.94 × 10^7^ cfu, respectively. The corresponding numbers for D39Δ*tatD* infected mice are 1.07 ± 0.23 × 10^6^, 8.54 ± 5.33 × 10^4^ and 5.40 ± 1.36 × 10^6^, respectively.

We next analyzed the lung pathology in mice infected intraperitoneally with D39 or D39Δ*tatD* pneumococci. The lung was excised at 24 h time point, sections were stained with haematoxylin-eosin and scored for the degree of lung pathology. The lung infected with the wildtype D39 strain showed higher histology score (2.67 ± 0.33%) characterized by elevated intra-alveolar and mild interstitial inflammation as compared to lung from D39Δ*tatD* infected mice (1.33 ± 0.33%) which showed absence of intra-alveolar inflammation but had mild interstitial inflammation (Fig. 8b,c). This is consistent with the higher bacterial load in mice infected with D39 compared to its isogenic *tatD* deficient mutant at 24 h post infection.

To gain insight in the role played by TatD in pneumococcal virulence BALB/c mice (12 per group) were infected with D39 or D39Δ*tatD* intraperitoneally and the mice were monitored for symptoms every 12 h. At dose 10^4^ cfu per mouse 10 out of 12 mice succumbed by 72 h in the group inoculated with D39 strain (Fig. 8d upper panel) with a median survival time of 42 h. In the group that was given D39Δ*tatD* mutant (10^4^ cfu per mouse) all the mice survived. When the inoculum was increased to 10^5^ cfu per mouse we observed that mice infected with D39Δ*tatD* pneumococci (median survival time 48 h) showed delayed onset of symptoms compared to mice infected with wildtype pneumococci (median survival time 36 h; Fig. 8d lower panel). In summary, these data indicate that *tatD* deficient *S. pneumoniae* is compromised in virulence and its ability to cause pathogenesis in the murine sepsis model.

We also performed the Kaplan-Meier survival analysis wherein the inoculum was administered to mice intranasally. The number of animals that survived at the end of 336 h in the group that was given D39 and D39Δ*tatD* (dose = 5 × 10^8^ cfu per mouse) were 7 and 8, respectively (Supplementary Fig. S6). At 10^9^ cfu per mouse all mice in both groups that were given wildtype and *tatD* deficient strain turned moribund at 12 h time point. Unexpectedly, we did not find any statistically significant difference between the wildtype and *tatD* deficient strain in the intranasal infection model.

## Discussion

The innate arm of the immune system serves as the first line of defense against invading microbial pathogens. Neutrophils play a key role in eliminating the microbe during early stages of the infection. Neutrophils tackle microbes primarily using three strategies. The first one involves phagocytosing the microbe followed by intracellular killing of the microbe. The second strategy pertains to release of granules containing antimicrobial factors from neutrophils. The third strategy involves trapping the bacteria followed by NET mediated killing^31^. Although most microbial pathogens are cleared by the antimicrobial activities of neutrophils, several bacterial pathogens have evolved strategies to evade the killing mechanism of neutrophils.

We observed that *S. pneumoniae* secretes/releases nuclease(s). Our in-gel DNase activity data suggests the presence of upto 3 DNases in the secretome of *S. pneumoniae* strain D39 (Fig. 1f). We observed that the TatD containing middle band was of lighter intensity than the other two bands. For the in-gel DNase activity assay the secretome was initially resolved on a SDS-PAG during which all the proteins would have got denatured followed subsequently with renaturation steps where a fraction of proteins would have regained their native structure. The ability and degree to which a protein will regain native structure and activity is likely to vary from protein to protein. It may be noted that rTatD expressed in *E. coli* as inclusion bodies and had to be renatured in small steps to obtain enzymatically active protein. Thus, the intensity of the bands observed in the in-gel DNase activity assay may not reflect the DNase activity of the native form of the nuclease. In experiments where live pneumococci were used TatD appears to be a significant contributor towards the hydrolysis of human NETs (Fig. 7). The putative nucleases corresponding to the topmost and bottommost band can in principle contribute to NET degradation but we have not experimentally quantitated their relative contribution. Nucleases have also been reported to be secreted/released from other human pathogens where they serve different functions. For example, *S. aureus* is reported to secrete a nuclease toxin that plays a crucial role in targeting bacterial competitors^62^. The secreted DNase, Mpn491, from *Mycoplasma pneumoniae*, has been demonstrated to play a key role in evading NET-mediated bactericidal activity^63^. McGugan *et al*. have reported the presence of secretory nucleases in the human protozoan pathogen *Entamoeba histolytica* and the authors hypothesize that the nucleases digest host nucleic acids to fulfill its requirement for pyrimidine and purine precursors for growth and survival^64^.

Nucleases typically require divalent cations for optimal activity. Unlike most nucleases, TatD showed some “basal” DNase activity in the absence of exogenously added divalent cations. We used commercially available top quality water from a highly reliable source for preparing the required reagents and performing the picogreen assay. From the presented data we cannot rule out that trace amount of divalent cations were indeed absent in our assay system. Among the cations tested Ca^2+^, Co^2+^ and Mn^2+^ increased the DNase activity a little but to a statistically significant level (Fig. 4a–c) whereas Zn^2+^ and Mg^2+^ inhibited the activity (Fig. 4d,e). Like in the case of TatD, the DNA hydrolase activity of PfTatD from *P. falciparum* is reported to be inhibited by Mg^2+ ^^65^. Bacterial pathogens are likely to be exposed to different pH and ion concentration in the host during infection. Blood pH in rats has been documented to get altered during pneumococcal sepsis^66^. Both Ca^2+^ and Mg^2+^ are required for optimal activity of Streptococcal wall-anchored nuclease^36^. The extracellular Nuclease A from *S. agalactiae* is inactive in the absence of divalent cations. Mg^2+^ is required for its optimal activity. Mn^2+^ and Zn^2+^ could substitute for Mg^2+ ^^35^. The cell-wall anchored DNase (SpnA) present in *S. pyogenes* has a preference for Mg^2+^ and Ca^2+^ as cofactor for its activity. SpnA is completely inactive in the absence of Mg^2+ ^^34^. As a part of this study we also tested the substrate preference and specificity of rTatD. We observed that rTatD degraded *S. pneumoniae* genomic DNA, eukaryotic DNA and supercoiled double-stranded plasmid DNA efficiently (Fig. 4g). rTatD digested linearized double-stranded plasmid DNA with lower efficiency. Based on the ability of rTatD to digest supercoiled double-stranded plasmid DNA we concluded that rTatD is a non-specific endodeoxyribonuclease. Broad substrate specificity has been reported for other nucleases as well. For example, the surface attached extracellular nuclease, Nuc2, from *S. aureus* has the ability to digest salmon sperm DNA, *S. aureus* genomic DNA, single-stranded DNA and double-stranded plasmid DNA^67^. Morita *et al*. demonstrated that cell wall-anchored nuclease of *S. sanguinis* digested multiple forms of DNA derived from bacteriophage ΦX174, NET DNA and human RNA^36^. Similarly, Nuclease A from *S. agalactiae* also showed lack of substrate specificity. Nuclease A degraded chromosomal DNA, single- and double-stranded PCR product, plasmid DNA and RNA^35^.

In the various bacterial pathogens it has been observed that the nuclease is either surface exposed or secreted/released. *S. sanguinis* encodes a surface exposed nuclease that contributes to its survival when trapped in NETs^36^. SsnA from *Streptococcus suis* degrades NETs thereby evading anti-microbial action of NETs^58^. In *M. tuberculosis*, the extracellular nuclease Rv0888 is secreted by the bacteria^68^. All these nucleases possess a signal peptide. Unlike the above-mentioned nucleases, TatD lacks a signal peptide. Remarkably, we found TatD to be associated with the EV fraction (Fig. 5). The only other instance where a DNase activity was documented to be associated with the extracellular outer membrane vesicles was from *P. gingivalis*^41^. At this stage it is not clear whether TatD is on the surface of EVs or present in its lumen. It is intriguing how TatD would degrade NETs in the latter case. Based on this study and published data on EndA it follows that *S. pneumoniae* has an EV-associated DNase (TatD) and a membrane-anchored nuclease. A secreted nuclease like TatD could potentially be more effective in degrading NETs than a membrane-associated nuclease like EndA that has limited access to NETs. The cell wall association of EndA implies that it can only degrade NETs in the immediate vicinity of the trapped microbial pathogen. Pathogens that express more than one extracellular nuclease have been reported. At least two extracellular nucleases are present in *S. suis*. *V. cholerae* evades NETs with the help of two nucleases^69^. Having membrane bound and secreted nucleases could be an advantage. Moreover, two extracellular DNases may have widely different requirements for optimal activity (e. g. pH optimum) and may be expressed during different stages of the bacterial growth curve or in different niches in the host^70^. Interestingly, in our *in vivo* experiments we observed D39Δ*tatD* strain was impaired in its virulence properties compared to the wildtype strain in the sepsis model (Fig. 8d). By contrast, the impairment in the virulence properties of D39Δ*tatD* was not evident in the intranasal infection model (Supplementary Fig. S6).

Neutrophils play a key role in the clearance of *S. pneumoniae* from the host. Nucleases have been demonstrated to degrade DNA present in NETs thereby helping bacterial pathogens in escaping from NETs^32^. For example, the DNase SdaI has been shown to confer resistance to *S. pyogenes* against killing by neutrophils^71^. We observed that rTatD efficiently degraded NETs (Fig. 6). NET degradation by D39∆*tatD* mutant was lower compared to wildtype strain (Fig. 7). TatD could thus play a key role in aiding NET entrapped *S. pneumoniae* escape and spread to distant sites in the host. TatD represents a novel evasion strategy deployed by *S. pneumoniae* to overcome a key non-phagocytic mechanism to clear extracellular pneumococci. The authors speculate that pneumococcal strains that express higher levels of TatD are likely to be more virulent as they can escape from NETs more efficiently. This notion however needs to be tested experimentally.

Our studies with the murine sepsis model indicated that deletion of TatD (i) resulted in lower bacterial burden in lungs, blood and spleen, (ii) compromised the ability of pneumococci to cause lung pathology and (iii) delayed death in mice (Fig. 8) suggesting that TatD is a virulence factor, and is required for full virulence. Unlike in the sepsis model the difference between the Kaplan-Meier survival profile for D39 and D39Δ*tatD* were not statistically significant in the intranasal infection model. Mice infected with *S. pneumoniae* deficient in *endA* showed delayed onset of severe disease and a higher survival rate than the wildtype strain^20^. Similar observations have been documented from other human pathogens. DNase Sda1 from *S. pyogenes* has been demonstrated to contribute to bacterial virulence in a mouse model of necrotizing fasciitis^71^. It was shown that the *S. agalactiae* extracellular nuclease NucA is required for full infection in mouse model as nucA deficient strain is cleared rapidly from the lung tissue and showed decreased mortality in infected mice^35^. Similar observations were reported for *Streptococcus equi* mutants deficient in extracellular nucleases ENuc and 5Nuc^72^.

In summary, the present study demonstrated that the pneumococcal secretome possesses DNase activity that is conserved across serotypes. The activity is proteinase K and heat sensitive. In-gel DNase activity assay followed by mass spectrometry led to the identification of a novel DNase, TatD which belongs to the TatD_DNase family. The purified rTatD showed DNase activity. Biochemical studies showed that TatD is a non-specific endodeoxyribonuclease. TatD is associated with the EV compartment. The capability of *S. pneumoniae* to degrade human NETs is compromised in the absence of *tatD in vitro*. Our data indicates that the DNase activity of TatD contributes to escape of *S. pneumoniae* from NETs. The deletion of TatD resulted in decreased bacterial burden, less severe lung tissue pathology and higher survival rate of mice than wildtype *S. pneumoniae* in a murine model of sepsis. TatD is thus a virulence factor. TatD can be a potential target for developing drugs against pneumococcal infections. The role of TatD during human infection remains to be elucidated. It would be interesting to characterize the role of potentially novel nucleases corresponding to the other two bands observed in the in-gel DNase activity assay in the biology of *S. pneumoniae*.

## Electronic supplementary material

Supplementary Information
