# Supplementary material for: A novel extracellular vesicle-associated endodeoxyribonuclease helps Streptococcus pneumoniae evade neutrophil extracellular traps and is required for full virulence
Source: Sci Rep. 2018 May 22;8:7985. doi: 10.1038/s41598-018-25865-z (PMC5964101; doi:10.1038/s41598-018-25865-z)
Supplement: Supplementary file 1 — Supplementary Information [file 41598_2018_25865_MOESM1_ESM.pdf]

SUPPLEMENTARY INFORMATION FOR

A novel extracellular vesicle-associated endodeoxyribonuclease helps *Streptococcus pneumoniae* evade neutrophil extracellular traps and is required for full virulence

Hina Jhelum, Hema Sori and Devinder Sehgal*

Molecular Immunology Laboratory, National Institute of Immunology, New Delhi, India

*Corresponding Author: devinder@nii.ac.in

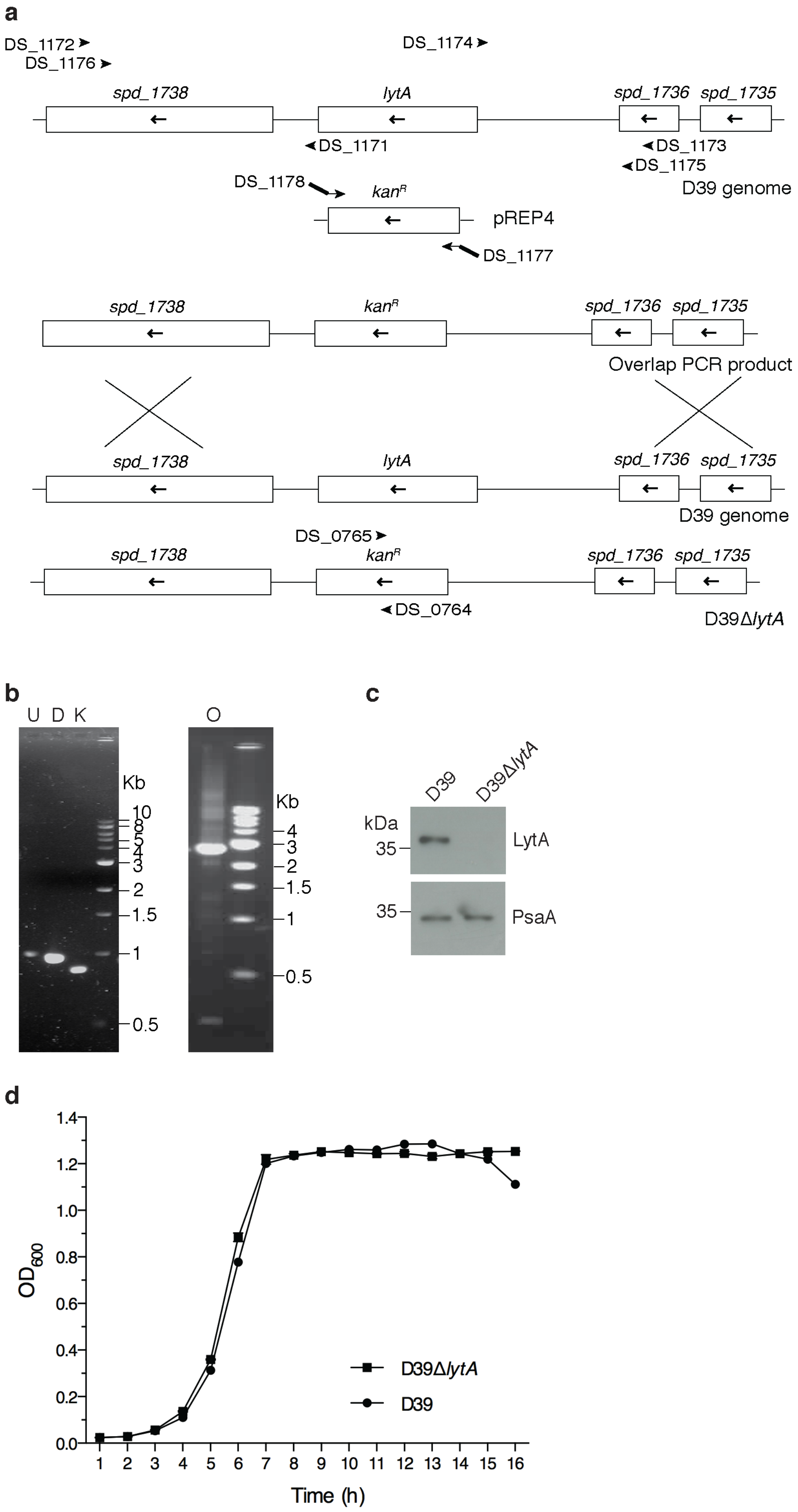
a
DS_1172
DS_1176
DS_1174
spd_1738
lytA
spd_1736
spd_1735
DS_1171
DS_1173
DS_1175
D39 genome
DS_1178
kanR
pREP4
DS_1177
Overlap PCR product
D39 genome
DS_0765
DS_0764
D39ΔlytA
b
U D K
O
Kb
c
D39
D39ΔlytA
kDa
35
LytA
PsaA
d
OD600
Time (h)
D39ΔlytA
D39

Supplementary Figure S1: Construction and molecular characterization of pneumococcal mutant strain D39Δ*lytA*. (a) Schematic representation of how D39Δ*lytA* mutant was generated using in-frame gene replacement mutagenesis. The gene of interest (*lytA*) and its neighbouring genes are shown in the schematic diagram as a rectangular box with the arrow (←) depicting their transcriptional orientation in the annotated *S. pneumoniae* strain D39 genome sequence. The PCR primers used for amplifying the upstream and downstream flank from the D39 genomic DNA, and the kanamycin resistance gene cassette (*kan*^{R}) from *E. coli* plasmid pREP4 are represented as rightward (➤) and leftward (➤) arrowheads. The details regarding the primers are provided in Supplementary Table S1. The primers with overhangs are depicted with an additional slanting line (⟍). The double crossover recombination events with the overlap PCR products are shown. (b) Ethidium bromide stained agarose gels showing amplicon corresponding to the upstream flank ('U', 1,005 bp), downstream flank ('D', 970 bp) and *kan*^{R} cassette ('K', 852 bp). The three PCR fragments were fused to form an overlap PCR product ('O', 2,621 bp). The molecular mass marker (in Kb) is shown on the right of the gel. The gel image on the left is uncropped. The full-length gel for the panel on the right is shown in Supplementary Figure S7. (c) Immunoblotting of whole cell lysates of D39 and D39Δ*lytA* with polyclonal sera raised against rLytA (refer to Supplementary Figure S4). Expected band of size 36.5 kDa corresponding to LytA was observed in D39 and was absent in D39Δ*lytA*. The blot was reprobed for PsaA (34.5 kDa) to confirm equal loading in lanes. Molecular mass marker (in kDa) is shown on the left. The full-length blots are presented in Supplementary Figure S7. (d) Growth kinetics was performed following the protocol reported by Mellroth *et al.* D39 (filled circle, ●) and D39Δ*lytA* (filled square, ■) in THY medium. In stationary phase the optical

density of the wildtype strain starts dipping at 16 h time point while that of the mutant remains stable.

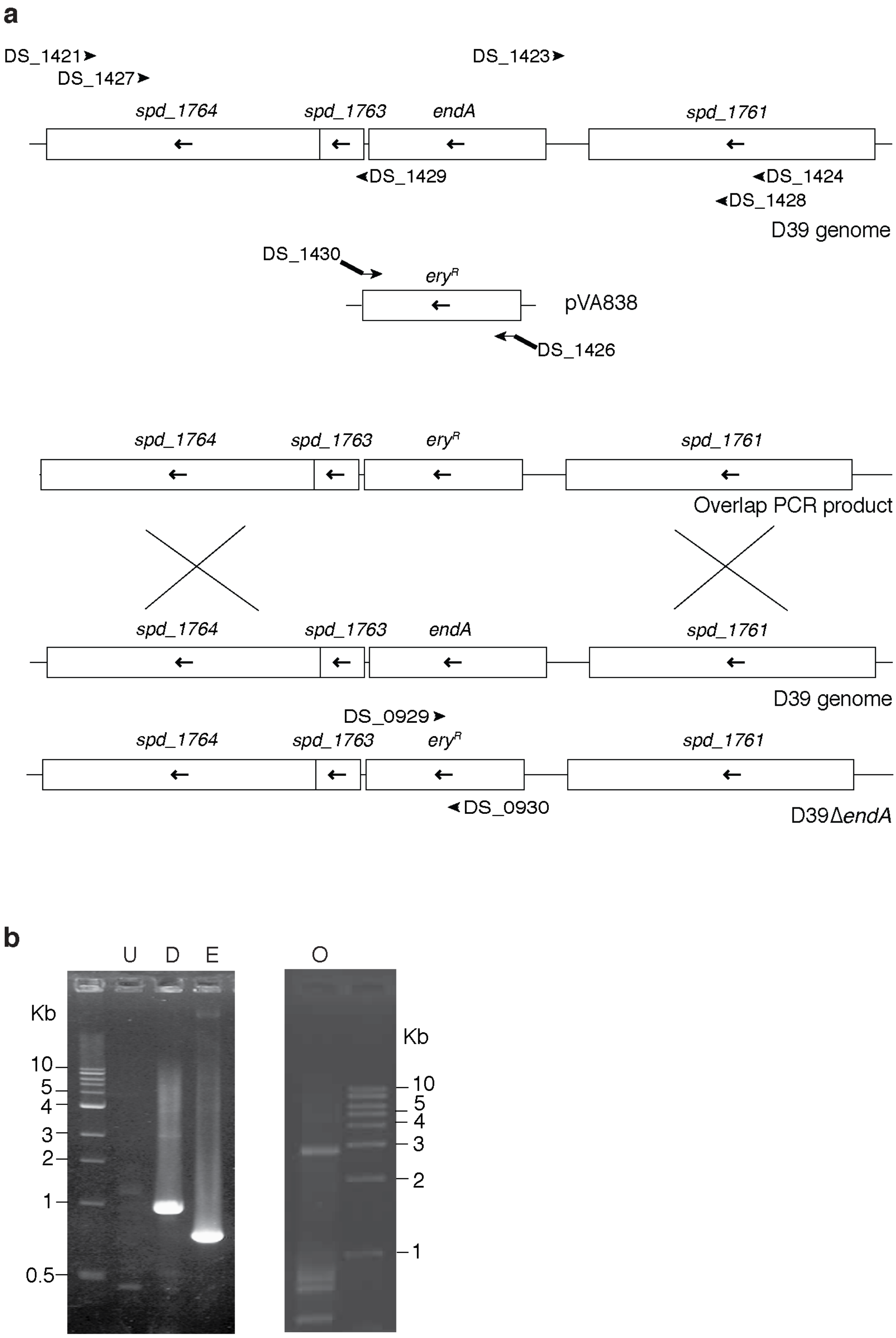

Supplementary Figure S2: Construction and molecular characterization of *S. pneumoniae* mutant strain D39Δ*endA*. (a) The overall strategy used for construction of D39Δ*endA* mutant was similar to that detailed in Supplementary

Figure S1. The erythromycin resistance gene cassette (*ery*^{R}) was amplified from plasmid pVA838 (obtained from American Type Culture Collection). The primers used for generating a strain deficient in *endA* are given in Supplementary Table S1. (b) Agarose gels with amplicons corresponding to upstream region (‘U’, 1,172 bp) and downstream region of *endA* (‘D’, 1,001 bp), and *ery*^{R} cassette (‘E’, 790 bp). These three fragments were fused to form an overlap PCR product (‘O’, 2,700 bp). For other details refer to legend to Supplementary Figure S1. The molecular mass marker (in Kb) is indicated. The full-length gels are presented in Supplementary Figure S7. For genetic confirmation of D39Δ*endA* refer to Supplementary Figure S3d.

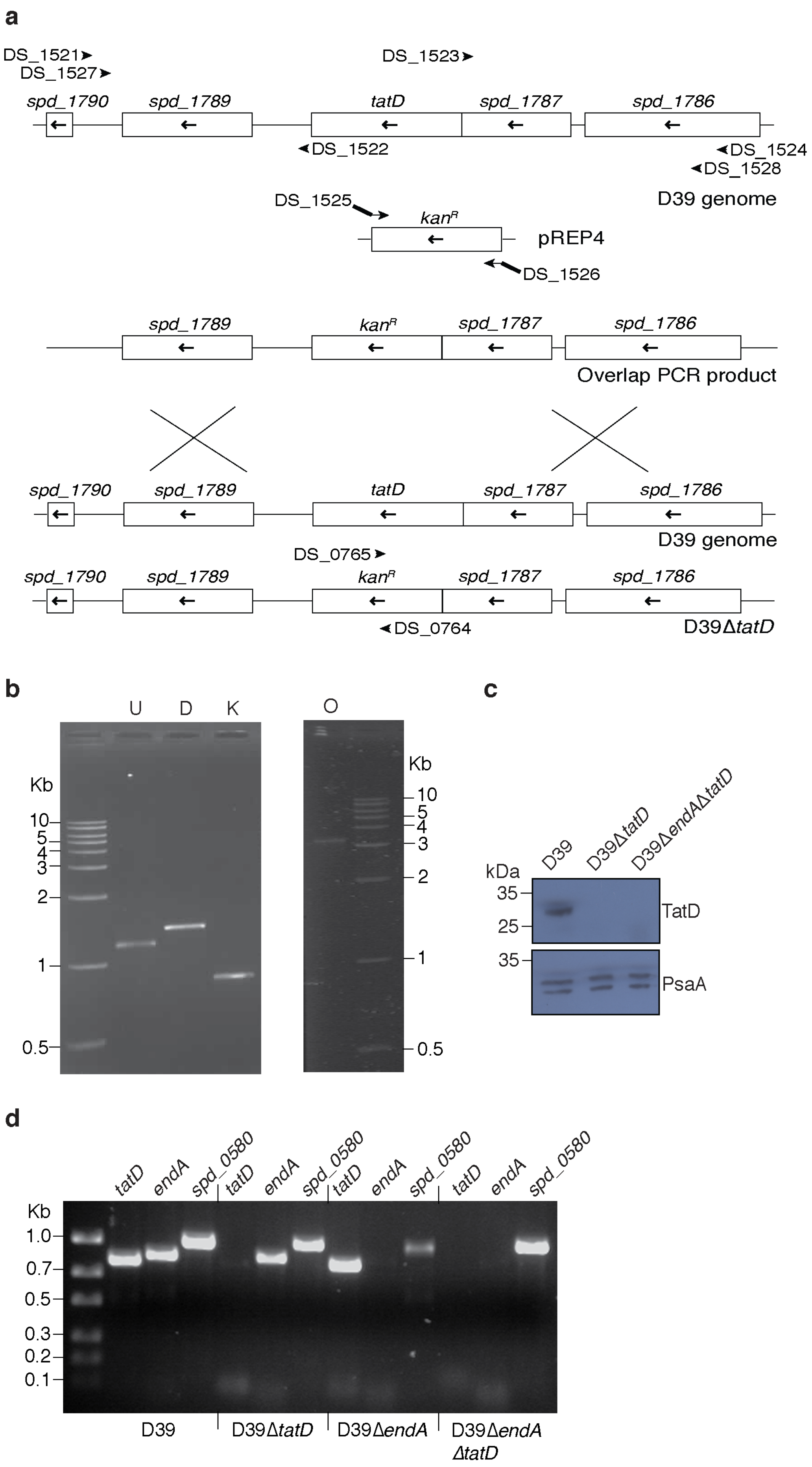
a
DS_1521
DS_1527
DS_1523
spd_1790
spd_1789
tatD
spd_1787
spd_1786
DS_1522
DS_1524
DS_1528
D39 genome
DS_1525
kanR
pREP4
DS_1526
spd_1789
kanR
spd_1787
spd_1786
Overlap PCR product
spd_1790
spd_1789
tatD
spd_1787
spd_1786
D39 genome
DS_0765
spd_1790
spd_1789
kanR
spd_1787
spd_1786
DS_0764
D39ΔtatD
b
U
D
K
O
Kb
10
5
4
3
2
1
0.5
c
kDa
D39
D39ΔtatD
D39ΔendAΔtatD
35
25
TatD
35
PsaA
d
tatD
endA
spd_0580
Kb
1.0
0.7
0.5
0.3
0.2
0.1
D39
D39ΔtatD
D39ΔendA
D39ΔendA ΔtatD

Supplementary Figure S3: Construction and molecular characterization of pneumococcal mutant strain D39Δ*tatD*. (a) Graphic representation for the strategy used for the construction of D39Δ*tatD* mutant using in-frame gene replacement mutagenesis. For details refer to legend to Supplementary Figure S1. (b) PCR amplification of upstream ('U', 1,223 bp) and downstream ('D', 1,400 bp) flanks of *tatD* from D39 genomic DNA along with kanamycin resistance gene cassette (kan^R) from pREP4 plasmid ('K', 844 bp). The three PCR products were fused to form an overlap fragment ('O', 3,200 bp). The molecular mass marker (in Kb) is indicated. For generating D39Δ*endA*Δ*tatD* double knockout the gene coding for TatD was replaced in D39Δ*endA* mutant with kanamycin resistance gene cassette using in-frame gene replacement mutagenesis. The full-length gels are shown in Supplementary Figure S7. (c) Characterization of D39Δ*tatD* and D39Δ*endA*Δ*tatD* by immunoblotting cell lysates of D39, D39Δ*tatD* and D39Δ*endA*Δ*tatD* with polyclonal sera generated against rTatD. Expected band of size 28 kDa corresponding to TatD was observed in D39 lysate and was absent in D39Δ*tatD* and D39Δ*endA*Δ*tatD*. The blot was reprobed for PsaA (34.5 kDa) to confirm equal loading. The full-length blots are shown in Supplementary Figure S7. (d) Genetic characterization of D39Δ*tatD* and D39Δ*endA*Δ*tatD* was done by PCR amplifying *tatD* and *endA* from genomic DNA*.* Glucokinase (*spd_0580*) was taken as a positive control. The primers used for PCR-based confirmation of these deletion mutants are provided in Supplementary Table S1. The resultant PCR products were resolved on an ethidium bromide containing agarose gel (0.8%). The uncropped gel image is shown in Supplementary Figure S7. The molecular mass marker (in Kb) is shown.

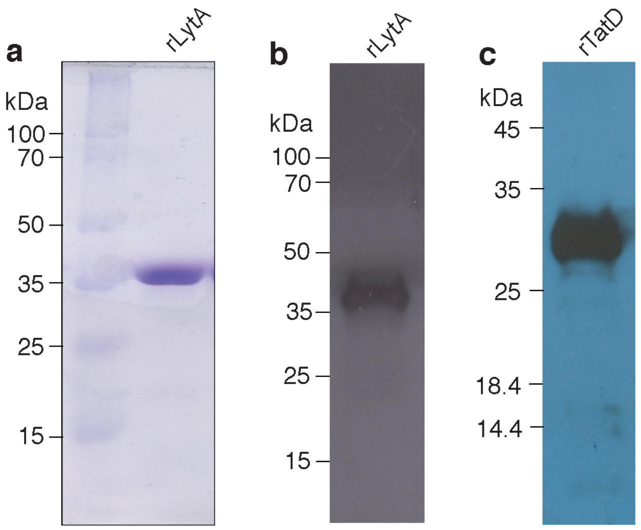

Supplementary Figure S4: Anti-rLytA antibody is specific. (a) SDS-PAGE analysis of purified recombinant LytA (rLytA). The *E. coli* strain expressing LytA was a kind gift from Peter Mellroth, Karolinska Institute, Sweden. rLytA was purified using the protocol described by Mellroth *et al*. [1]. Briefly, *E. coli* Rosetta 2 containing recombinant construct pET21d-*lytA* was grown in LB medium. Cells were induced with IPTG (2 mM) when the culture attained an OD_{600} of 0.5. LytA was purified from the soluble fraction using diethylaminoethyl sepharose fast flow ion exchange chromatography (GE Healthcare, UK). Immunoblot analysis of rLytA (b) and rTatD (c) with mouse anti-rLytA and anti-rTatD polyclonal antibody, respectively. The molecular mass markers (in kDa) used are shown to the left of each panel. The uncropped gel/ blots are presented in Supplementary Figure S7.

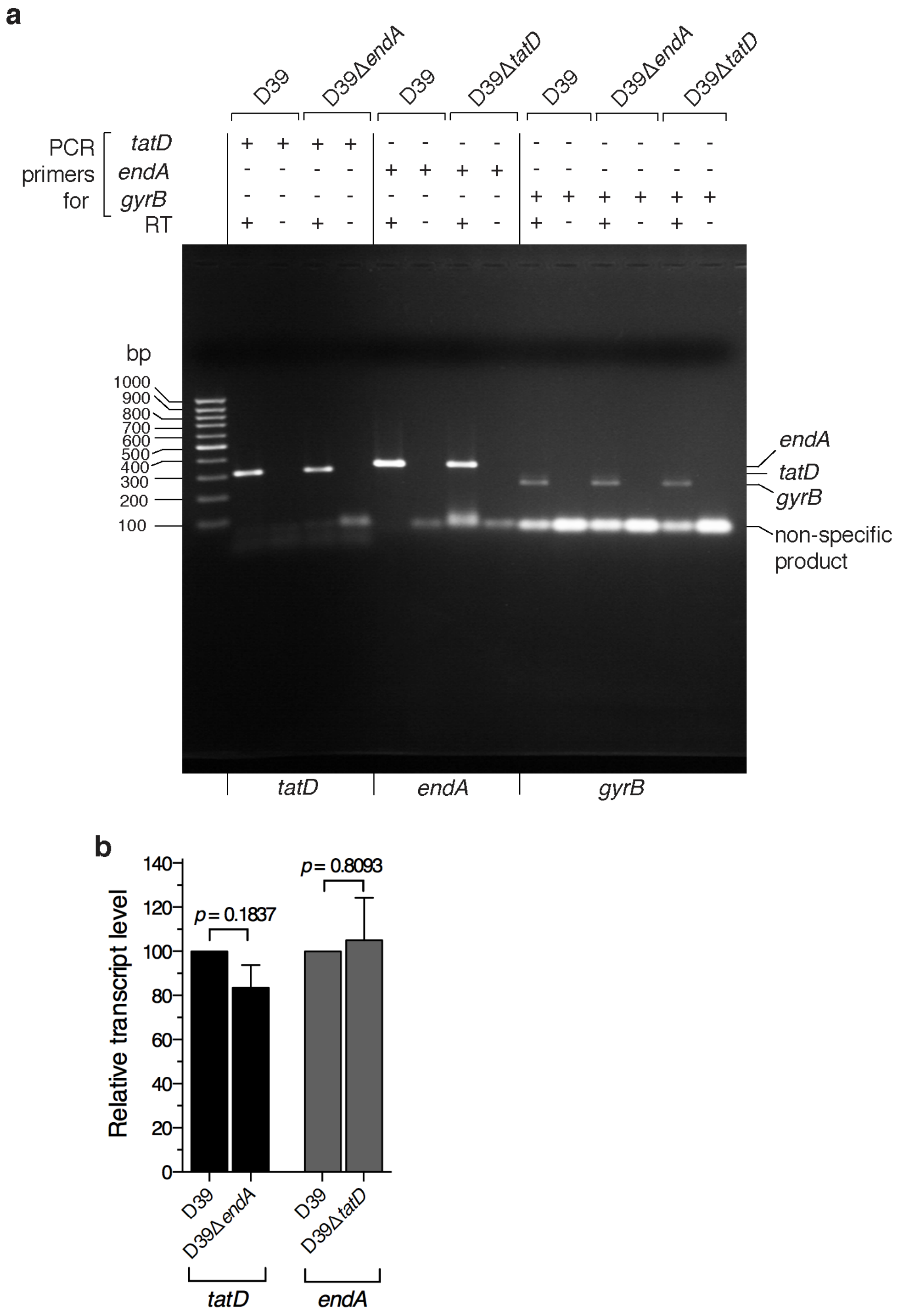

Supplementary Figure S5: Semi-quantitative reverse-transcriptase PCR to assess the levels of *tatD* and *endA* transcripts in pneumococcal strains D39Δ*endA* and D39Δ*tatD*, respectively relative to that in the wildtype D39 strain. Total RNA from pneumococcal strains D39, D39Δ*tatD* and D39Δ*endA* was isolated using a commercially available kit (RNeasy Mini kit, Qiagen) following the

manufacturer's protocol. Briefly, *S. pneumoniae* strains D39, D39Δ*tatD* and D39Δ*endA* were grown to mid-logarithmic phase (OD_{600} = 0.4-0.5) in THY medium and pneumococci were harvested from 3 ml culture (2 × 10^8) by centrifuging at 5,000 × g at 4°C for 10 min. The pellet was resuspended in 100 µl TE-lysozyme buffer (lysozyme = 3 mg/ml) and incubated at room temperature for 10 min. RLT buffer (350 µl) containing β-mercaptoethanol (10 µl/ml) was added, vortexed vigorously and the sample was centrifuged at 20,000 × g for 2 min. Absolute ethanol (250 µl) was added to the supernatant, mixed thoroughly and loaded on a RNeasy Mini Spin column. The column was centrifuged at 20,000 × g for 15 sec and the flow through was discarded. The column was washed with RW1 buffer (700 µl) at 20,000 × g for 15 sec followed by two more washes with RPE buffer (500 µl) for 15 sec and 2 min, respectively. The blank column was centrifuged at 20,000 × g for 1 min. The column was transferred to a fresh collection tube and total RNA was eluted in 30 µl RNase-free water. To remove potential DNA contamination, 3 µg total RNA (quantitated using Nanodrop, Thermo Scientific, USA) was digested with 1 U RNase-free TURBO DNase I (Ambion, Invitrogen) in a 25 µl reaction mixture containing 1× TURBO DNase buffer. The reaction mixture was incubated at 37°C for 30 min. The reaction was terminated by adding 2.5 µl of DNase inactivation reagent and incubated further at room temperature for 5 min. The mixture was centrifuged at 20,000 × g for 5 min and the supernatant was collected. The total RNA obtained was used to assess the transcript levels of *tatD* and *endA* by reverse transcriptase-PCR (RT-PCR).

First strand cDNA was synthesized from total RNA using a commercially available RT-PCR kit (QuantiTect Reverse transcription kit, Qiagen) using gene specific primers for *tatD*, *endA* and *gyrB* (DNA gyrase B subunit, *spd_0709*; used as internal reference gene) as detailed in Supplementary Table S1. To remove any

possible genomic DNA contamination, a 12 µl reaction mixture containing total RNA (500 ng) along with 2 µl of gDNA wipeout and RNase-free water was set up, incubated at 42°C for 2 min and transferred on ice immediately. A mix containing 1 U reverse transcriptase, 4 µl of 5× reverse transcriptase buffer and 1 µl of 10 µM primer mix containing primers for first strand cDNA synthesis of *tatD*, *endA* and *gyrB* transcripts was added to the above-mentioned mix. The sample was incubated at 42°C for 30 min followed by incubation at 95°C for 3 min to inactivate the enzyme. A control reaction was run wherein reverse transcriptase was omitted (- RT control).

The semi-quantitative RT-PCR methodology used for the analysis was adapted from Chen *et al*. [2]. We used *gyrB*, a known constitutively expressed housekeeping gene as an internal reference to normalize the expression of *tatD* and *endA* transcripts. In preliminary experiments, a five-fold dilution series of the first strand cDNA was used to arrive at the linearity range of the exponential phase of PCR amplification. To PCR amplify *tatD*, 5 µl first strand cDNA product (at a dilution in the linear range of PCR amplification) was used as template in a 50 µl PCR reaction mix containing 1× Taq DNA polymerase buffer, 200 µM dNTP mix, 0.75 µM *tatD* sense and antisense primer and 1 U Taq DNA polymerase. Touchdown hot start PCR was performed for amplifying the first strand cDNA. The PCR cycling parameters used were an initial denaturation step of 94°C for 3 min followed by denaturation at 94°C for 30 sec, annealing at 65°C for 30 sec and extension at 72°C for 1 min. This was run for 5 cycles with a 2°C drop in the annealing temperature per cycle. The next 25 cycles were run with denaturation at 94°C for 30 sec, annealing at 55°C for 30 sec and extension at 72°C for 1 min followed by a final extension at 72°C for 10 min. The PCR was performed in a similar manner to amplify *endA* and *gyrB* first strand cDNAs. PCR amplicons corresponding to *tatD* (314 bp), *endA* (348

bp) and *gyrB* (248 bp) from pneumococcal strains D39, D39Δ*tatD* and D39Δ*endA* were resolved on an ethidium bromide containing agarose gel (a). Molecular weight marker (in bp) is shown on the left. The data presented is representative of three independent experiments. The gel image shown is uncropped. RT, reverse transcriptase. (b) Densitometric analysis of the expression level of *endA* and *tatD* transcripts in D39Δ*tatD* and D39Δ*endA* strain, respectively relative to that in D39 was performed using ImageJ. The expression level of *tatD* transcript in a given total RNA preparation from D39 and D39Δ*endA* was normalized with the expression level of *gyrB* in the respective strains. The same procedure was followed to normalize the expression level of *endA* in D39 and D39Δ*tatD* strains. To facilitate comparison of expression level across three total RNA preparations the expression level of *tatD* in wildtype D39 strain was arbitrarily assigned a value of 100 and the relative expression of *tatD* in D39Δ*endA* mutant was calculated. The expression level of *endA* in D39Δ*tatD* strain was normalized in a similar manner. The error bars represent mean ± sem of three experiments performed using three independent preparations of total RNA from the wildtype and mutant strains. Unpaired two-tailed Student's *t* test was used for statistical analysis.

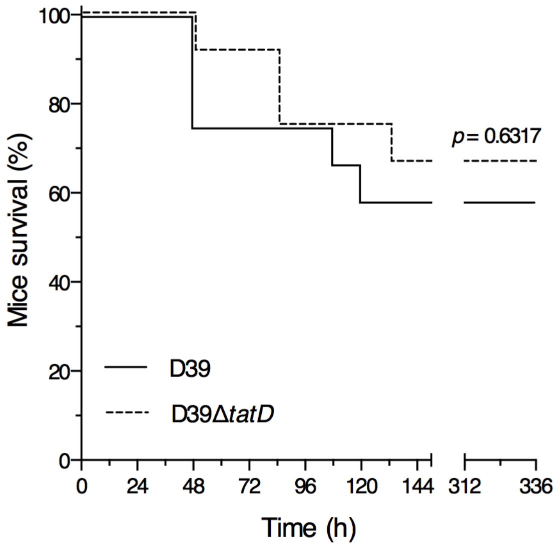

Supplementary Figure S6: Mice survival analysis. Kaplan-Meier survival analysis of 6-8 weeks old female BALB/c mice (12 per group) anaesthetized with ketamine-xylazine (100 and 10 mg/kg, respectively in 100 μl) were infected intranasally with 5 × 10^8 cfu (in 20 μl) per mouse of *S. pneumoniae* D39 or D39Δ*tatD* strain. The health of mice was monitored and scored as described in the *Materials and Methods*. This experiment was repeated with a higher dose (10^9 cfu per mouse). We observed that all mice (12 mice per group) turned moribund at 12 h time point irrespective of whether they were administered wildtype or *tatD* deficient strain (data not shown). Statistical analysis was done using Log-rank (Mantel-Cox) test.

Fig. 1b

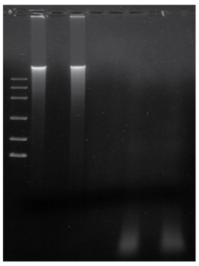

Fig. 1c

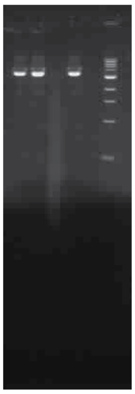

Fig. 1d

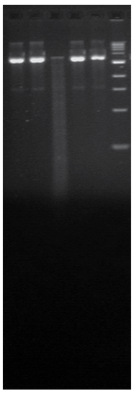

Fig. 1e

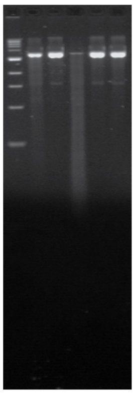

Fig. 1f

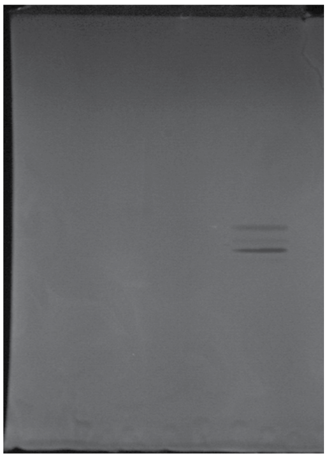

Fig. 1g

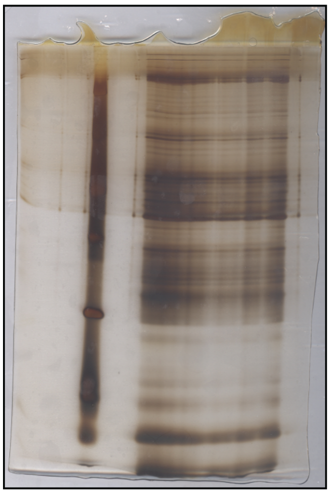

Fig. 3a

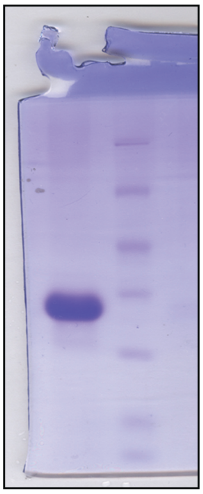

Fig. 3b

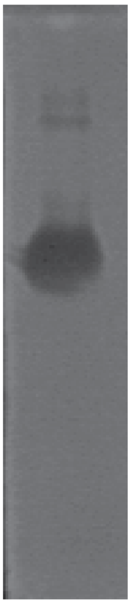

Fig. 4g
left panel

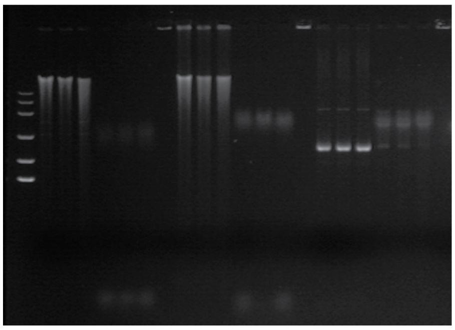

Fig. 4g
right panel

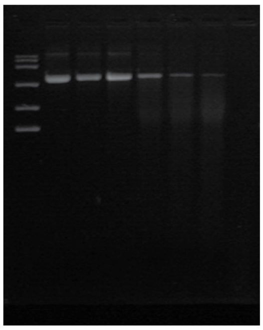

Fig. 5a
TatD

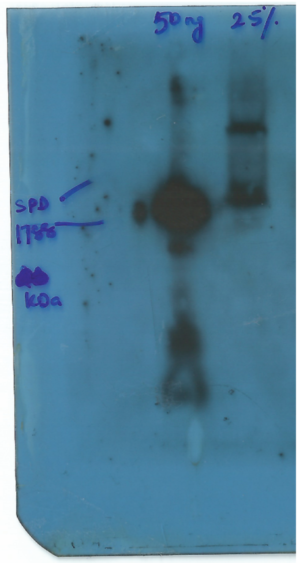

Fig. 5a
Pneumolysin

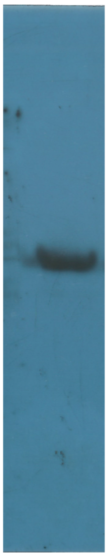

Fig. 5b

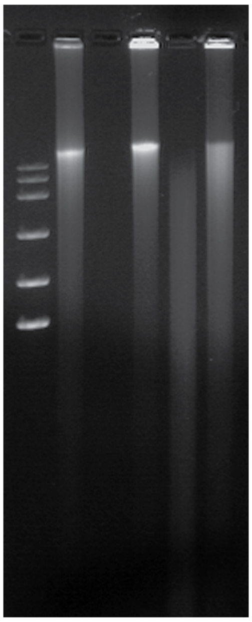

Supplementary Fig. S1b
right panel

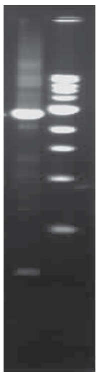

Supplementary Fig. S1c
LytA

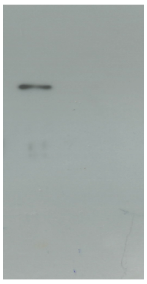

Supplementary Fig. S1c
PsaA

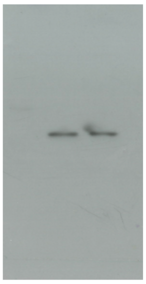

Supplementary Fig. S2b
left panel

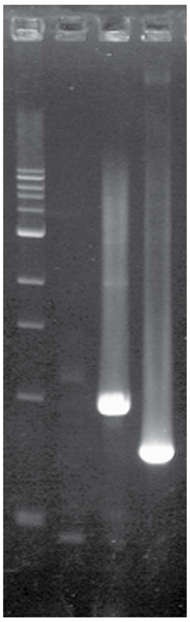

Supplementary Fig. S2b
right panel

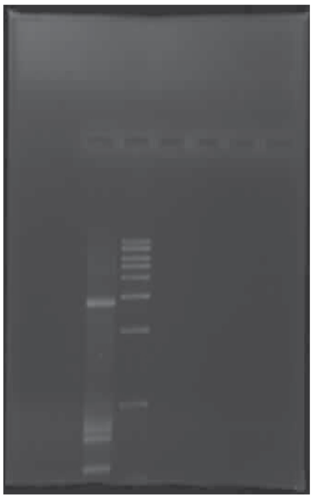

Supplementary Fig. S3b
left panel

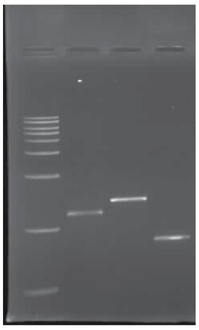

Supplementary Fig. S3b
right panel

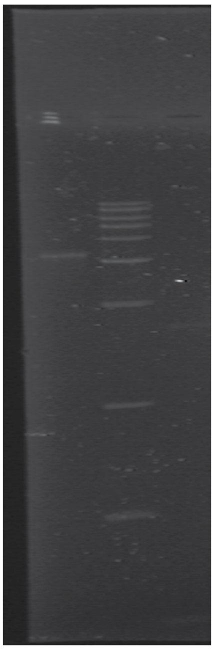

Supplementary Fig. S3c
TatD

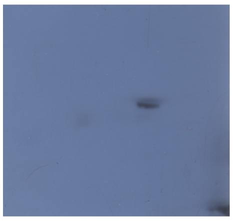

Supplementary Fig. S3c
PsaA

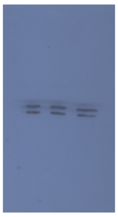

Supplementary Fig. S3d

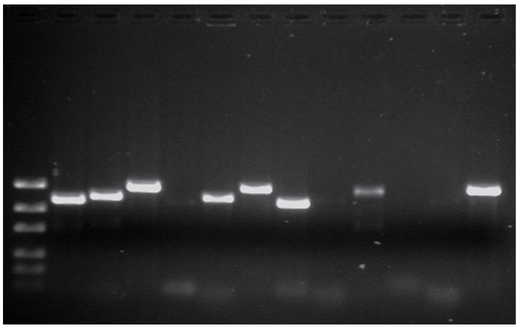

Supplementary Fig. S4a

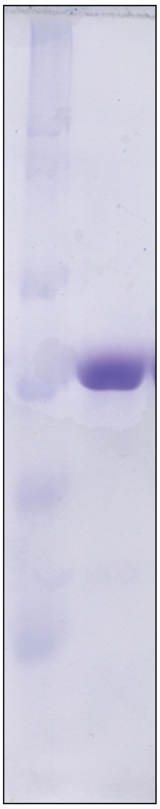

Supplementary Fig. S4b
rLytA

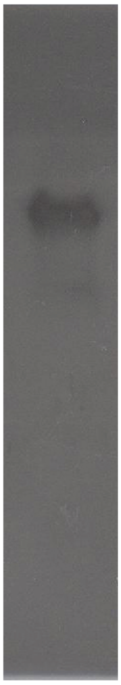

Supplementary Fig. S4c
rTatD

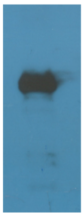

Supplementary Figure S7: Full-length gels and blots. The figure numbers are indicated above the image.

References

[1] Mellroth, P. *et al.* LytA, major autolysin of *Streptococcus pneumoniae*, requires access to nascent peptidoglycan. *J Biol Chem* **287**, 11018-11029, doi:10.1074/jbc.M111.318584 (2012).

[2] Chen, L., Segal, D. M. & Mash, D. C. Semi-quantitative reverse-transcriptase polymerase chain reaction: an approach for the measurement of target gene expression in human brain. *Brain Res Brain Res Protoc* **4**, 132-139 (1999).

Supplementary Table S1. Primers used in this study for generation and PCR-based confirmation of pneumococcal deletion mutants, and reverse transcriptase-PCR

**Primer**	**Primer Sequence (5' to 3')**	**Primer binding site and directionality**[a]	**Colony PCR**[b]	**Nucleotide sequencing**[c]
**D39Δ*lytA***				
DS_0764	tga tgc aat gcg gcg gct g	*kan*^{R} resistance cassette/ leftward	+	+
DS_0765	cga gta cgt gct cgc tcg a	*kan*^{R} resistance cassette/ rightward	+	+
DS_1171	att cta ctc ctt atc aat taa aac aac	Upstream flank/ leftward		
DS_1172	ggc ttg atg agt tca att gta tct a	Upstream flank/ rightward/ external	+	+
DS_1173	tga ctg ata cca taa aac tga ccc	Downstream flank/ leftward/ external	+	+
DS_1174	taa tgg aat gtc ttt caa atc aga ac	Downstream flank/ rightward		
DS_1175	gat ttc ctc aac cat cct ata cag tg	Downstream flank/ leftward/ internal		
DS_1176	att agt gcc cag acg gca gct cg	Upstream flank/ rightward/ internal		
DS_1177	*ctg ttc tga ttt gaa aga cat tcc att a*tc aga aga act cgt caa gaa ggc	*kan*^{R} resistance cassette/ leftward/ with overhang[d]		
DS_1178	*gag ttg ttt taa ttg ata agg agt aga ata* tgc ttg aac aag atg gat tgc ac	*kan*^{R} resistance cassette/ rightward/ with overhang[d]		
**D39Δ*endA***				
DS_0929	cat tcc gct ggc agc tta ag	*ery*^{R} resistance cassette/ rightward	+	+
DS_0930	aag tgg ttt ttg aaa gcc atg c	*ery*^{R} resistance cassette/ leftward	+	+
DS_1421	gtg gtt gta cga ttg gta gcc	Upstream flank/ rightward/ external	+	+

DS_1423	aag ata agc cta aac tcc tat gt	Downstream flank/ rightward		
DS_1424	tcc tta tcc cca tca taa acc g	Downstream flank/ leftward/ external	+	+
DS_1426	*tga cat agg agt tta ggc tta tct t*tt att tcc tcc cgt taa ata ata g	*ery*^{R} resistance cassette/ leftward/ with overhang[d]		
DS_1427	tag tca gac agc tgg tta cat cg	Upstream flank/ rightward/ internal		
DS_1428	gct tga cta tca tcc tga ata tcc	Downstream flank/ leftward/ internal		
DS_1429	ctg tct tgt ttt ttt gtt cat att t	Upstream flank/ leftward		
DS_1430	*aga aat atg aac aaa aaa aca aga cag* atg aac aaa aat ata aaa tat tct caa	*ery*^{R} resistance cassette/ rightward/ with overhang[d]		
**D39Δ*tatD***				
DS_0764	tga tgc aat gcg gcg gct g	*kan*^{R} resistance cassette/ leftward	+	+
DS_0765	cga gta cgt gct cgc tcg a	*kan*^{R} resistance cassette/ rightward	+	+
DS_1521	ttc tat caa aaa tca gta gga ac	Upstream flank/ rightward/ external	+	+
DS_1522	ctc ttc taa cct cgt ttt cta t	Upstream flank/ leftward		
DS_1523	tga aag aga gaa ttt ccc aag tt	Downstream flank/ rightward		
DS_1524	ctc tta atg ctt gct tcc aca	Downstream flank/ leftward/ external	+	+
DS_1525	*gga tag aaa acg agg tta gaa gag* atg ctt gaa caa gat gga ttg c	*kan*^{R} resistance cassette/ rightward/ with overhang[d]		
DS_1526	*ata act tgg gaa att ctc tct ttc* atc aga aga act cgt caa gaa g	*kan*^{R} resistance cassette/ leftward/ with overhang[d]		
DS_1527	cta gaa tag tac acc tct gtt tct aa	Upstream flank/ rightward/ internal		
DS_1528	ctt gta aac acg ata gat aat cat taa a	Downstream flank/ leftward/ internal		

**PCR-based confirmation of pneumococcal deletion mutants**		
DS_1371	ccc ccc gga tcc atg agt caa aag att att ggg att	Sense primer for *spd_0580*
DS_1372	ccc ccc ctg cag tta ttg caa tac aag tga tgc tg	Antisense primer for *spd_0580*
DS_1401[e]	ccc ccc gga tcc atg att ttt gat aca cat aca cac tt	Sense primer for *tatD*
DS_1402[e]	ccc ccc ctg cag tta ctt gct gtc caa tcc aaa aat	Antisense primer for *tatD*
DS_1411	ccc ccc gga tcc atg aac aaa aaa aca aga cag aca c	Sense primer for *endA*
DS_1412	ccc ccc ctg cag tta ctg agt tac agt tac ttc tcc	Antisense primer for *endA*
**RT-PCR primers[f]**		
DS_1691	ata aac tca cga cga g	*gyrB* primer for cDNA synthesis
DS_1692	gtg tca agg ttg gaa gcg ag	Sense primer for *gyrB*
DS_1693	cat caa cat atc aaa gat ttt atc t	Antisense primer for *gyrB*
DS_1929	cag tcg ttg cta ccg	*tatD* primer for cDNA synthesis
DS_1930	gtc cat acc cgt gat gcg c	Sense primer for *tatD*
DS_1931	tca gcg ata aag tcg acc ac	Antisense primer for *tatD*
DS_1932	acc gat taa ggc ata g	*endA* primer for cDNA synthesis

DS_1933	agt ccc aaa acc aat ctt agt c	Sense primer for *endA*
DS_1934	ctt tag att ctt gac ctg atg c	Antisense primer for *endA*

[a]The directionality (rightward or leftward) of the primers used for generating pneumococcal gene deletion mutants is shown in Supplementary Figures S1-S3.

[b]Primer used for colony PCR.

[c]Primer used for nucleotide sequencing.

[d]The italicized portion in the primer sequence represents overhang.

[e]A BamHI and PstI site (underlined) was engineered in primers DS_1401 and DS_1402, respectively to facilitate cloning of *tatD* amplicon in *E. coli* expression vector pQE-30 Xa.

[f]Semi-quantitative reverse-transcriptase PCR based assessment of expression levels of *tatD* and *endA* transcripts relative to that of housekeeping gene *gyrB*.

Supplementary Table S2. Mass spectrometry based identification of proteins

Locus[a]	Description	Score	Coverage[b]	No. of Unique peptides
SPD_0526	Fructose-1, 6-bisphosphate aldolase, class II	1763.57	60.41	23
SPD_1463	ABC transporter, substrate binding lipoprotein	265.44	27.18	6
SPD_0913	Hypothetical protein	202.86	59.57	17
SPD_0150	ABC transporter, substrate-binding protein	192.15	47.83	11
SPD_1246	Glucosamine-6-phosphate deaminase	113.82	75.32	11
SPD_1823	Glyceraldehyde-3-phosphate dehydrogenase, type I	92.59	12.84	3
SPD_0726	Purine nucleoside phosphorylase	81.20	33.83	6
SPD_0580	Glucokinase	67.80	20.38	4
SPD_0196	50S ribosomal protein L2	61.27	23.10	6
SPD_0542	Dipeptidase PepV	57.31	14.38	4
SPD_2043	Secreted 45 kDa protein	51.04	27.30	7
SPD_0151	Lipoprotein	36.61	39.08	8
SPD_0868	Foldase protein PrsA	25.38	24.28	5
SPD_1287	Thioredoxin-disulfide reductase	24.67	18.48	4
SPD_0772	1-phosphofructokinase, putative	23.19	10.23	2
SPD_1404	Triosephosphate isomerase	20.08	21.03	3
SPD_0672	Peptidyl-prolyl cis-trans isomerase, cyclophilin-type	14.95	19.10	3
SPD_0328	Glucose-1-phosphate thymidylyltransferase	14.64	12.46	3
SPD_1984	Hypersensitive-induced reaction protein 4	13.25	6.35	1
SPD_1412	GTP-sensing transcriptional pleiotropic repressor CodY	12.73	17.56	4
SPD_0852	Dihydroorotate dehydrogenase, catalytic subunit	12.28	8.33	2
SPD_1725	Probable transcriptional regulatory protein	10.94	10.08	2
SPD_1078	L-lactate dehydrogenase	10.39	9.15	2
SPD_0730	Purine nucleoside phosphorylase DeoD-type	9.75	13.56	3

SPD_2068	Serine protease	8.02	9.92	2
SPD_0851	Dihydroorotate dehydrogenase, electron transfer subunit	7.78	10.90	3
SPD_0970	Methionine aminopeptidase, type I	7.36	5.24	1
SPD_0352	DNA-binding response regulator	5.48	8.10	1
SPD_1776	Pur operon repressor PurR	4.62	11.27	2
SPD_2037	Cysteine synthase A	4.54	5.23	1
SPD_0665	Dihydroorotate dehydrogenase A	3.86	5.47	1
SPD_0442	CTP synthase	3.69	4.11	1
SPD_2015	33 kDa chaperonin	3.10	3.79	1
SPD_1788	Hydrolase, TatD family protein	2.76	7.00	1
SPD_0262	PTS system, mannose/ fructose/ sorbose family protein, IID component	2.45	3.96	1
SPD_1196	Adapter protein MecA	2.45	5.71	1
SPD_0558	Cell wall-associated serine protease PrtA	2.18	0.47	1
SPD_1402	Non-heme iron-containing ferritin	1.86	3.49	1
SPD_0980	Ribose-phosphate pyrophosphokinase	1.80	4.08	1

Mass spectrometry based identification of proteins present in the gel slice excised from a silver stained preparative polyacrylamide gel corresponding to the middle band with DNase activity (refer to Fig. 1f and g).

[a]The locus name is as per the annotated genome sequence of the pneumococcal strain D39.

[b]Percentage of total protein sequence covered by experimentally detected peptides.

Supplementary Table S3. TatD alleles, allele frequency and percent identity

Allele[a]	Pneumococcal strain (genome sequence identifier)	Relative allele frequency (%)	Identity (%)[b]
1	D39 (NC_008533.1), TIGR4 (NC_003028.3), OXC141 (NC_017592.1), SPN034183 (NC_021028.1), SPN994038 (NC_021026.1), SPN994039 (NC_021005.1)	6 (18.18)	100
2	Taiwan 19F-14 (NC_012469.1), SP64 (NZ_CP018138.1), SP61 (NZ_CP018137.1), ST556 (NC_017769.2), SWU02 (NZ_CP018347.1), TCH8431/ 19A (NC_014251.1)	6 (18.18)	99.61
3	Hungary 19A-6 (NC_010380.1)	1 (3.03)	99.61
4	670-6B (NC_014498.1)	1 (3.03)	99.61
5	G54 (NC_011072.1), SP49 (NZ_CP018136.1)	2 (6.06)	99.22
6	A66 (NZ_LN847353.1), 70585 (NC_012468.1), gamPNI0373 (NC_018630.1)	3 (9.09)	99.22
7	P1031 (NC_012467.1)	1 (3.03)	99.22
8	A45 (NC_018594.1)	1 (3.03)	98.83
9	NCTC7465 (NZ_LN831051.1), SNP034156 (NC_021006.1)	2 (6.06)	98.83
10	NT_110_58 (NZ_CP007593.1)	1 (3.03)	98.83
11	INV104 (NC_017591.1)	1 (3.03)	98.44
12	JJA (NC_012466.1), KK0981 (AP017971.1)	2 (6.06)	98.44
13	CGSP14 (NC_010582.1), INV200 (NC_017593.1)	2 (6.06)	98.44
14	SPN033038 (NC_021004.1), SPN032672 (NC_021003.1)	2 (6.06)	98.05

15	AP200 (NC_014494.1)	1 (3.03)	98.05
16	ATCC 700669 (NC_011900.1)	1 (3.03)	98.05

The 33 pneumococcal strains for which complete genome sequence is publically available (accessed on September 29, 2017) were analyzed for the presence of homologue of TatD from *S. pneumoniae* (strain R6 was excluded as it is a descendant of strain D39 which was included in the analysis).

[a]The amino acid sequence of TatD from strain D39 was aligned using MacVector software with its homologues from the other 32 strains for which complete genome sequence is available. The alleles were identified and numbered 1 through 16 arbitrarily.

[b] The amino acid identity was calculated for each allelic group relative to the amino acid sequence of allele 1.
